# Effect of a Multifactorial Intervention on Retinopathy in People With Type 2 Diabetes: A Secondary Analysis of the J-DOIT3 Randomized Clinical Trial

**DOI:** 10.1001/jamaophthalmol.2025.3819

**Published:** 2025-10-23

**Authors:** Takayoshi Sasako, Kohjiro Ueki, Kengo Miyoshi, Kana Miyake, Tomohisa Aoyama, Yukiko Okazaki, Naoki Ishizuka, Toshimasa Yamauchi, Mitsuhiko Noda, Takashi Kadowaki

**Affiliations:** 1 Department of Diabetes and Metabolic Diseases, Graduate School of Medicine, The University of Tokyo, Tokyo, Japan; 2 Diabetes Research Center, Japan Institute for Health Security, Tokyo, Japan; 3 Department of Metabolism and Endocrinology, Juntendo University Graduate School of Medicine, Tokyo, Japan; 4 Center for Digital Transformation of Healthcare, Graduate School of Medicine, Kyoto University, Kyoto, Japan; 5 Department of Diabetes, Metabolism and Endocrinology, Ichikawa Hospital, International University of Health and Welfare, Chiba, Japan; 6 Department of Endocrinology and Diabetes, Saitama Medical University, Saitama, Japan; 7 Toranomon Hospital, Tokyo, Japan

## Abstract

**Importance:**

Prevention of diabetic retinopathy is important to keep vision and quality of life.

**Objective:**

To examine the effects of an intensive multifactorial intervention and hypoglycemia on retinopathy in people with type 2 diabetes.

**Design, Setting, and Participants:**

J-DOIT3 (Japan Diabetes Optimal Integrated Treatment Study for 3 Major Risk Factors of Cardiovascular Diseases) is a multicenter, open-label, parallel-group randomized clinical trial that examined the efficacy of an intensified multifactorial intervention on cardiovascular outcomes and mortality in people with type 2 diabetes aged 45 to 69 years with hypertension and/or dyslipidemia. The study was conducted at 81 sites in Japan from June 2006 to March 2009, and data analysis was performed from September 2018 to August 2025.

**Interventions:**

Participants were randomly assigned to intensive therapy for glucose, blood pressure, and lipids or conventional therapy and were followed up for a median duration of 8.5 years.

**Main Outcomes and Measures:**

This study is a secondary analysis of retinopathy events of the secondary outcomes, composed of onset of retinopathy, progression of retinopathy, and loss of vision likely due to retinopathy.

**Results:**

Among 2540 total participants (5080 eyes) randomly assigned to intensive therapy or conventional therapy, mean (SD) age was 59.0 (6.3) years, and 965 participants (38.0%) were female. Intensive therapy was associated with a risk reduction in onset of retinopathy (hazard ratio [HR], 0.83; 95% CI, 0.70-0.98; *P* = .03) but not with progression of retinopathy (HR, 1.02; 95% CI, 0.70-1.49; *P* = .93). Hemoglobin A_1c_ (HbA_1c_) at 1 year after randomization was associated with onset (HR, 1.31; 95% CI, 1.13-1.51; *P* < .001), even after adjustment for baseline risk factors (ie, lower body mass index, longer duration of diabetes, higher fasting plasma glucose, higher blood pressure, and comorbid nephropathy), with no clear HbA_1c_ threshold observed. Moreover, compared with those without a hypoglycemic episode during the intervention, the risk of onset was higher in those with 0.5 hypoglycemic episodes per year or fewer (HR, 1.25; 95% CI, 1.02-1.53) and even higher in those with more than 1 hypoglycemic episode per year (HR, 1.85; 95% CI, 1.39-2.47).

**Conclusions and Relevance:**

This secondary analysis of the J-DOIT3 randomized clinical trial shows that in a randomized clinical trial setting, higher HbA_1c_ and nonsevere hypoglycemia are associated with higher risk of onset of retinopathy in people with type 2 diabetes, even when good glycemic management, with a very low incidence of severe hypoglycemia, is achieved, suggesting the importance of strict glycemic management without any hypoglycemia.

**Trial Registration:**

ClinicalTrials.gov Identifier: NCT00300976

## Key Points

**Question:**

How does strict multifactorial intervention, with good glycemic management and a low incidence of severe hypoglycemia, affect the onset and progression of retinopathy in people with type 2 diabetes?

**Findings:**

In this secondary analysis of the J-DOIT3 randomized clinical trial, a multifactorial intervention and glycemic management were associated with a risk reduction in onset but not progression of retinopathy. No clear hemoglobin A_1c_ threshold for onset of retinopathy was observed; hypoglycemia was associated with a higher risk of onset but not progression of retinopathy.

**Meaning:**

These findings support the increasing importance of strict glycemic management without any hypoglycemia to increase the chance of preventing retinopathy.

## Teaser

This secondary analysis of the J-DOIT3 randomized clinical trial examines the effects of an intensive multifactorial intervention and hypoglycemia on retinopathy in people with type 2 diabetes.

## Introduction

The goal of diabetes management is to achieve longevity and quality of life equivalent to those in people without diabetes by preventing onset or progression of diabetic vascular complications.^1^ Retinopathy is one of the major microvascular complications, which could even cause loss of vision and significantly impair quality of life. In previous landmark clinical trials, such as the DCCT (Diabetes Control and Complications Trial),^2^ the risk of retinopathy was reported to be reduced by improved glycemic management. However, insulin therapy was used in many of the participants, or even all of them.^2,3,4,5^ Thus, an important controversy remains, which matters in the prevention of retinopathy: improved glycemic management or glycemic management by raising circulating insulin level. Moreover, the benefit of glycemic management was not replicated by several other trials.^6,7,8^ Further, it remains unclear whether there exists a hemoglobin A_1c_ (HbA_1c_) threshold for retinopathy.^4,9,10,11^

To further complicate matters, acute improvement in glycemic management results in progression of preexisting diabetic retinopathy,^12^ although onset and progression were not clearly distinguished in some of the previous trials.^7,8,11,13^ Severe hypoglycemia was also reported to elevate the risk of onset,^14^ while intensive therapy was shown to be frequently associated with severe hypoglycemia in previous trials,^15^ even though sufficient glycemic management was not achieved (HbA_1c_ approximately 8%) in some of them. Thus, the relationship between glycemic management and retinopathy in people with good glycemic management without severe hypoglycemia remains to be precisely examined.

Additionally, evidence of retinopathy prevention by strict blood pressure management^16,17,18,19^ and lipid-lowering therapy using fibrates^5,18,20,21^ suggests the benefits of multifactorial intervention. Indeed, in the Steno-2 study, multifactorial intervention suppressed onset and progression of retinopathy in people with type 2 diabetes and microalbuminuria. However, over half of participants were treated with insulin, and still the glycemic management was insufficient (HbA_1c_ approximately 8%), and severe hypoglycemia was frequent.^13,22^ It also remains unclear which component of the intervention affected the risk of retinopathy. Moreover, the benefit was not replicated in the ADDITION-Europe (Anglo-Danish-Dutch Study of Intensive Treatment In People with Screen-Detected Diabetes in Primary Care) study, where the effects of a multifactorial intervention for newly diagnosed type 2 diabetes were examined.^22,23,24^

We recently reported the primary results of the J-DOIT3 (Japan Diabetes Optimal Integrated Treatment Study for 3 Major Risk Factors of Cardiovascular Diseases) study, a randomized clinical trial in which 2540 Japanese participants with type 2 diabetes were followed up for a median duration of 8.5 years.^25,26,27,28^ Major risk factors were improved beyond or almost equally to the targets recommended by clinical guidelines, even in the conventional therapy group, but they were further improved in the intensive therapy group, including HbA_1c_ (6.8% in the intensive therapy group vs 7.2% in the conventional therapy group). Notably, insulin therapy was used in less than 15% of the participants at the end of the intervention (14.1% vs 9.8%). Intensive therapy was associated with a nonsignificant risk reduction in the primary outcome, composed of myocardial infarction, stroke, revascularization, and all-cause mortality (hazard ratio [HR], 0.81). It was also associated with a significant risk reduction in retinopathy events (HR, 0.86), one of the secondary outcomes, composed of nonproliferative or proliferative retinopathy in healthy eye(s) at baseline, proliferative retinopathy in eye(s) with nonproliferative retinopathy at baseline, and loss of vision likely due to retinopathy. Notably, no loss of vision occurred in either group. Although the incidence of severe hypoglycemia was very low in both groups (0.6% vs 0.3%), the incidence of any hypoglycemia was higher in the intensive therapy group (41% vs 22%).^28^ Thus, the J-DOIT3 trial stands in clear contrast to the DCCT, where individuals with type 1 diabetes were treated with insulin, with less strict glycemic management in the conventional therapy group (HbA_1c_ approximately 9%) and frequent severe hypoglycemia, especially in the intensive therapy group (62 episodes per 100 person-years).^2^

Here, we describe a secondary analysis of retinopathy events as a prespecified secondary outcome in the J-DOIT3 study.^26,27,28^ We examined the effects of not only intensified multifactorial intervention, but also each component of treatment (eg, glycemic management) and hypoglycemia on the onset or progression of retinopathy.

## Methods

### Study Outline

The J-DOIT3 trial was a multicenter, open-label, parallel-group randomized clinical trial that examined the efficacy of an intensified multifactorial intervention on cardiovascular outcomes and mortality in type 2 diabetes (NCT00300976).^25,26,27,28^ In this trial, people with type 2 diabetes aged 45 to 69 years with hypertension and/or dyslipidemia and an HbA_1c_ of 6.9% or higher were registered at 81 clinical sites in Japan. Those with proliferative retinopathy according to the International Classification of Diabetic Retinopathy (ICDR) scale (equivalent to Diabetic Retinopathy Severity Scale [DRSS] levels 60-85)^29,30,31^ or blindness due to diabetic retinopathy were excluded. Participants were randomly assigned between June 2006 and March 2009 at a 1:1 ratio to either conventional therapy for treatment targets as specified in the Japanese guideline (such as HbA_1c_ <6.9%)^32^ or intensive therapy for stricter treatment targets (such as HbA_1c_ <6.2%).^27,28^ Fibrate treatment was discontinued in the intensive therapy group, because statin treatment to lower low-density lipoprotein cholesterol was one of the main components of intensive therapy, but the combination of a statin and a fibrate was contraindicated in Japan at that time. In the intensive therapy group, medications were intensified in a stepwise manner if participants failed to achieve the treatment targets in 3 to 6 months.

Dilated fundus examination was performed by an ophthalmologist at each institution once per year, and the diagnosis was classified according to the ICDR scale as no retinopathy (equivalent to DRSS level 10), nonproliferative retinopathy of any severity (DRSS levels 20-53), or proliferative retinopathy (DRSS levels 60-85).^29,30,31^ Diagnoses were reported through the electronic data capturing system. The intervention was continued until March 2016.^27,28^

All patients provided written informed consent. At study entry, participants in the intensive therapy group received a blood glucose meter, a sphygmomanometer, and a recordable accelerometer, whereas those in the conventional therapy group received a sphygmomanometer and a pedometer. The study protocol (Supplement 1) was approved by ethics committees at each participating institution, and the study was carried out in accordance with the principles of the Declaration of Helsinki as revised in 2008.^27,28^ This report follows the Consolidated Standards of Reporting Trials (CONSORT) reporting guidelines.^33^

### Outcomes

Retinopathy events were broken down into onset (ie, those occurring in participants without retinopathy at baseline) and progression (ie, those occurring in participants with nonproliferative retinopathy in ≥1 eye at baseline) in this study, as is summarized in eTables 1 and 2 in Supplement 2. Retinopathy events were determined by the End Point Assessment Committees, who were masked to the treatment groups, based on the reported diagnosis and other courses.^27,28^

### Adverse Events

In this trial, severe hypoglycemia and nonsevere hypoglycemia were among the prespecified adverse events and actively surveyed. At every visit, participants were asked about the presence or absence of these events by the physician in charge, and participants in the intensive therapy group reported self-monitored blood glucose. Occurrence of hypoglycemia was judged by the physician in charge by taking results of self-monitored blood glucose into account. Severe hypoglycemia was defined as hypoglycemia requiring someone else’s assistance or admission to hospital, which was judged based on the adverse event report made by the physician in charge. The collected adverse events were reviewed by the Safety Assessment Committee unmasked to the treatment groups.^27,28^

### Statistical Analysis

All confirmatory analyses of the outcomes were conducted in accordance with the prespecified statistical analysis plan and the intention-to-treat principle using SAS version 9.4 (SAS Institute). Time to first event of retinopathy events was summarized as a cumulative proportion with the Kaplan-Meier method and compared using the log-rank test. HRs representing the treatment effects were estimated by the Cox regression analysis, with treatment effect as the only covariate. With stratification factors and other important prognostic factors at baseline as covariates, the multivariable Cox regression analysis was conducted to assess sensitivity of the effects of intensive therapy on retinopathy events. These prespecified analyses followed those of the primary outcome and the subsequent subanalyses.^27,28,34,35^

The post hoc Cox regression was conducted as a landmark analysis using the factors applicable at 1 year after randomization (ignoring the events before 1 year).^34,35^ Participants were stratified by the presence or absence of retinopathy at baseline, by the treatment group, or by the yearly incidence of hypoglycemic episodes during the intervention period when needed. The cubic spline curve for the HR, with HbA_1c_ reference value of 6.9%, was estimated by the restricted cubic spline method for trend analysis.^36^ A 2-sided *P* value of less than .05 was deemed significant.

## Results

### Effects of Intensive Therapy on Retinopathy

The baseline characteristics in both groups are summarized in eTable 1 in Supplement 2.^28,34,35^ Among 2540 total participants (5080 eyes) randomly assigned to intensive therapy or conventional therapy, mean (SD) age was 59.0 (6.3) years, and 965 participants (38.0%) were female. No clear intergroup difference was observed in retinopathy at baseline.

**Figure 1.**
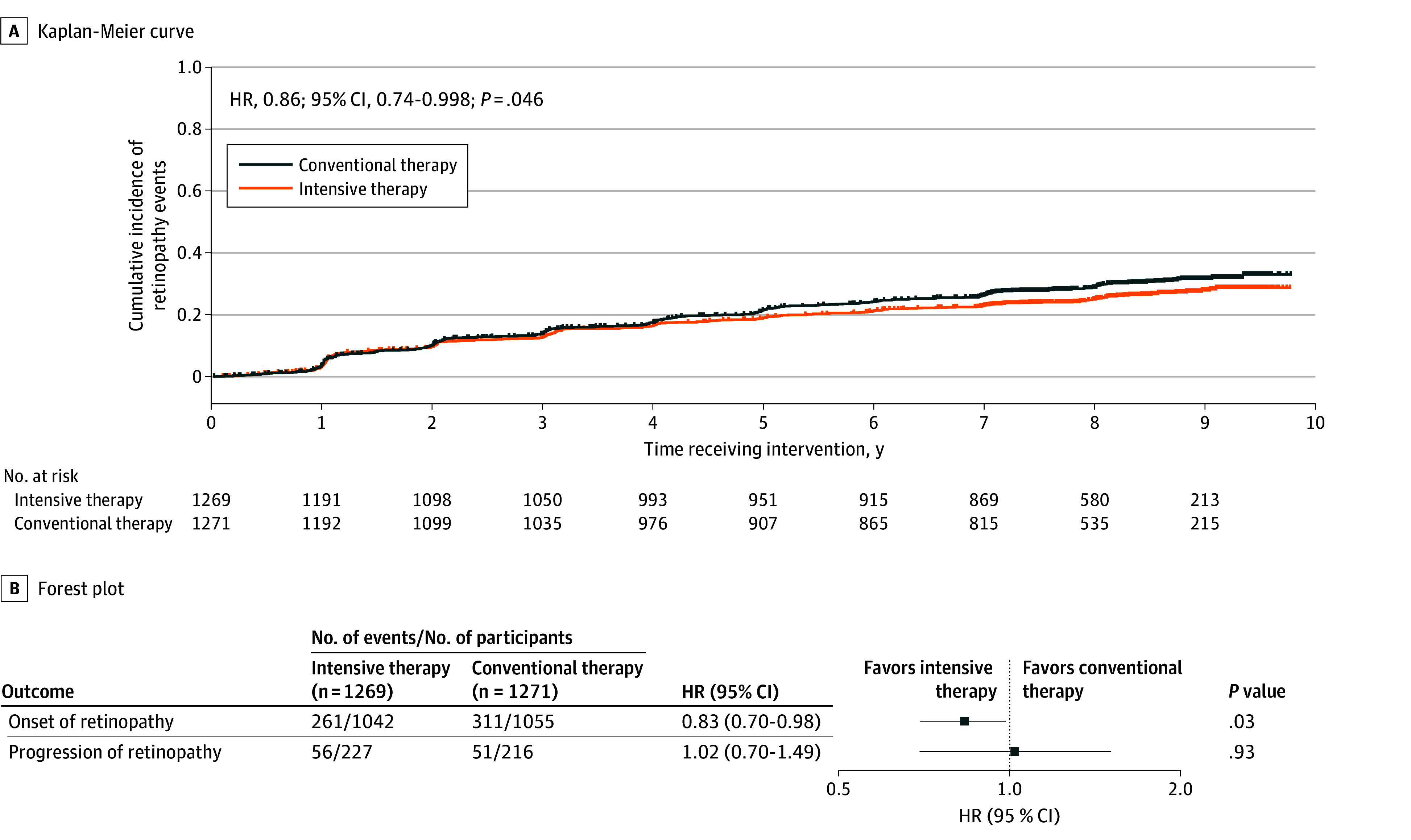
Cumulative Incidence of Retinopathy Events A, Kaplan-Meier curve to show the effect of intensive therapy on onset or progression of retinopathy. A total of 317 events were observed in 1269 participants in the intensive therapy group, and 362 events were observed in 1271 participants in the conventional therapy group. B, Forest plots to show the respective effects of intensive therapy on onset and progression of retinopathy, with raw *P* values presented. Participants were further stratified by the presence or absence of retinopathy at baseline. Bars represent 95% confidence intervals. HR indicates hazard ratio.

A total of 679 retinopathy events occurred as time to first events during the intervention period (317 in the intensive therapy group and 362 in the conventional therapy group). An examination of the cumulative incidence revealed that intensive therapy was associated with a significant risk reduction in retinopathy events compared with conventional therapy (HR, 0.86; 95% CI, 0.74-0.998; *P* = .046)^28^ (Figure 1A).

In a breakdown of the first retinopathy events (eTable 2 in Supplement 2), 261 events in the intensive therapy group (37.7 events/1000 person-years) and 311 events in the conventional therapy group (45.6 events/1000 person-years) were onset of retinopathy in participants without retinopathy at baseline, with intensive therapy associated with a risk reduction in onset (HR, 0.83; 95% CI, 0.70-0.98; *P* = .03) (Figure 1B). The rest, 56 events in the intensive therapy group (37.2 events/1000 person-years) and 51 events in the conventional therapy group (36.6 events/1000 person-years), were progression of retinopathy in participants with nonproliferative retinopathy in at least 1 eye at baseline, with intensive therapy associated with no risk reduction in progression (HR, 1.02; 95% CI, 0.70-1.49; *P* = .93) (Figure 1B).

Then, Cox regression analysis was performed using the prespecified baseline risk factors, finding that intensive therapy was associated with a risk reduction in retinopathy events after adjustment (HR, 0.85; 95% CI, 0.73-0.99; *P* = .04) (eTable 3 in Supplement 2). Similarly, intensive therapy was associated with a risk reduction in onset even after adjustment (HR, 0.83; 95% CI, 0.70-0.99; *P* = .03) (eTable 4 in Supplement 2). The risk of onset was associated with lower body mass index (BMI), higher systolic blood pressure, longer duration of diabetes, higher fasting plasma glucose, and the presence of albuminuria at baseline (eTable 4 in Supplement 2). By contrast, intensive therapy was not shown to be associated with progression, even after adjustment (HR, 1.01; 95% CI, 0.69-1.48; *P* = .97) (eTable 5 in Supplement 2), and the risk was associated with none of the other explanatory covariates evaluated.

### Effects of Components of Intensive Therapy on Onset of Retinopathy

Next, to determine the effect of each component of the treatment on onset of retinopathy, an additional Cox regression analysis was performed using the identified baseline risk factors and those found relevant at 1 year after randomization. After adjustment, HbA_1c_ at 1 year was associated with an increased risk of onset (HR, 1.31; 95% CI, 1.13-1.51; *P* < .001) (Table), but intensive therapy was not. It is thus suggested that the risk reduction in onset observed with intensive therapy could be attributable to improvements in glycemic management during the intervention.

**Table.** Multivariable Cox Regression Analysis of Onset of Retinopathy With Baseline Characteristics and Potential Risk Factors During the Intervention Period as Covariates^a^

Explanatory variable	Regression coefficient (95% CI)	*P* value
Treatment group, conventional vs intensive	1.02 (0.83-1.25)	.85
HbA_1c_ at baseline, <8.9% vs ≥8.9%	1.05 (0.81-1.37)	.71
Body mass index at baseline, <25 vs ≥25^b^	0.81 (0.67-0.99)	.04
Duration of diabetes at baseline, <10 y vs ≥10 y	1.44 (1.19-1.75)	<.001
Fasting plasma glucose at baseline, <180 mg/dL vs ≥180 mg/dL	1.24 (0.98-1.58)	.07
Systolic blood pressure at baseline, <130 mm Hg vs ≥130 mm Hg	1.37 (1.11-1.70)	.003
Urine ACR at baseline, <30 mg/g Cr vs ≥30 mg/g Cr	1.29 (1.04-1.60)	.02
HbA_1c_ at 1 y, %	1.31 (1.13-1.51)	<.001
Fasting plasma glucose at 1 y, mg/dL	1.00 (1.00-1.00)	.91
Systolic blood pressure at 1 y, mm Hg	1.00 (1.00-1.01)	.46

Abbreviations: ACR, albumin-creatinine ratio; HbA_1c_, hemoglobin A_1c_.

^a^ Multivariable Cox regression for onset of retinopathy occurring 1 year after randomization or later with allocation factors and prespecified factors at baseline and potential risk factors at 1 year after randomization as covariates. Covariates were available in 1738 participants without retinopathy at baseline, and 451 events were observed. The reference category of baseline characteristic is indicated in the left, and risk factors at 1 year are in continuous numbers.

^b^ Calculated as weight in kilograms divided by height in meters squared.

**Figure 2.**
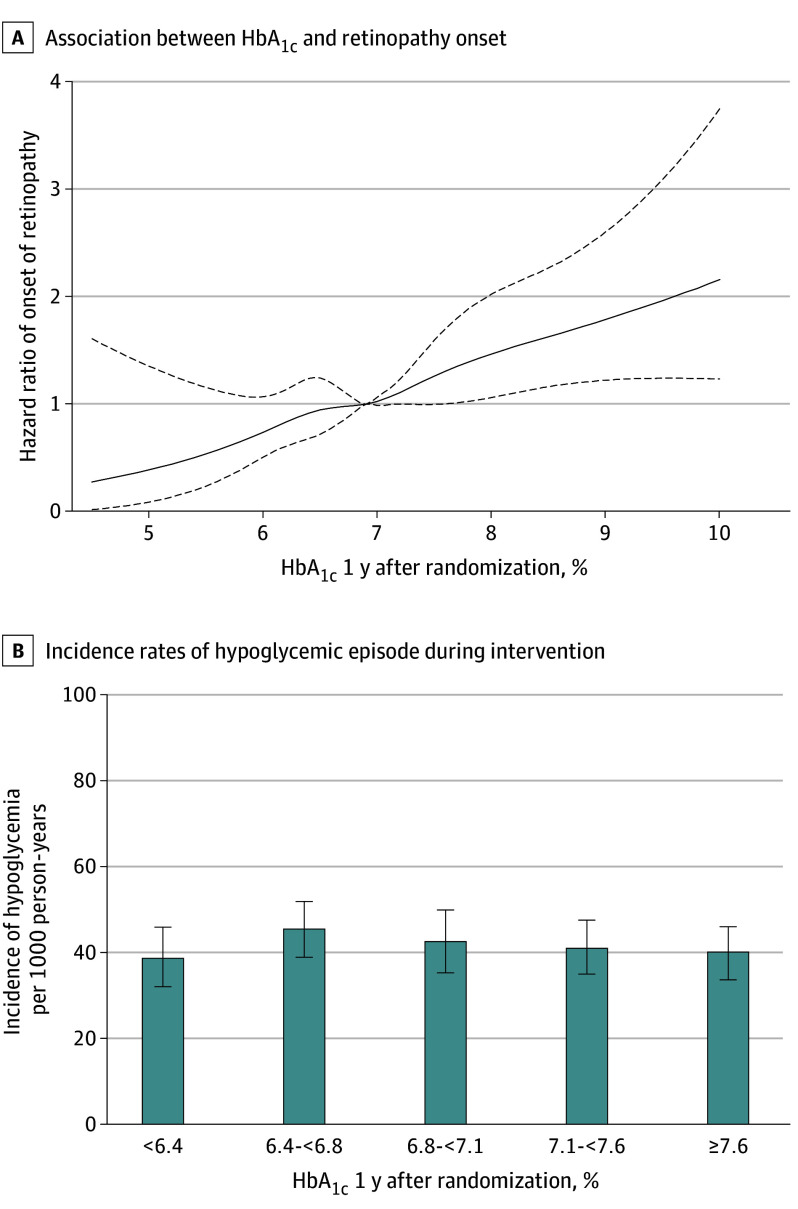
Glycemic Control and Onset of Retinopathy A, Cubic spline curve to show the association between hemoglobin A_1c_ (HbA_1c_) at 1 year after randomization and the incidence of onset of retinopathy during intervention. Participants without retinopathy at baseline were included, whereas participants were excluded in whom (1) HbA_1c_, fasting plasma glucose, or systolic blood pressure at 1 year after randomization was missing or (2) onset of retinopathy was reported within the first year after randomization, yielding 1738 participants and 451 events subject to the analysis. The reference HbA_1c_ value was 6.9%, the median at 1 year. The nodes were 6.0% (5th percentile), 6.5% (25th percentile), 6.9% (reference), 7.4% (75th percentile), and 8.5% (95th percentile). Dashed lines indicate 95% pointwise confidence intervals. B, Incidence rates of hypoglycemic episode during intervention per 1000 person-years. Participants in whom HbA_1c_ at 1 year after randomization was missing were excluded, and 2282 participants were stratified by the quintiles of HbA_1c_ at 1 year (6.4%, 6.8%, 7.1%, 7.6%). Bars represent 95% confidence intervals.

Moreover, restricted cubic spline analysis revealed that HbA_1c_ at 1 year after randomization was associated with the risk of onset in a linear manner, with no clear threshold (Figure 2A).

Improving glycemic management could be accompanied by an increase in hypoglycemia. We stratified participants by the quintiles of HbA_1c_ at 1 year and examined the incidence of hypoglycemic episode(s). Indeed, the incidence was shown to be almost unaffected by HbA_1c_ at 1 year and about 40 per 1000 person-years (Figure 2B). Moreover, the proportion of those experiencing at least a single hypoglycemic episode was higher in the participants with a 1% or greater reduction in HbA_1c_ during the first year (eTable 6 in Supplement 2), with a significant intergroup difference. These data suggest that better glycemic management could be achieved without an elevated risk of hypoglycemia, but that careful attention should be paid when blood glucose is improved rapidly.

### Effects of Hypoglycemia on Retinopathy

Lastly, given that hypoglycemia was more frequent in the intensive therapy group and that it is one of the risk factors for retinopathy, we examined the association between the yearly incidence of (mostly nonsevere) hypoglycemia (eTable 7 in Supplement 2) and onset or progression of retinopathy.

**Figure 3.**
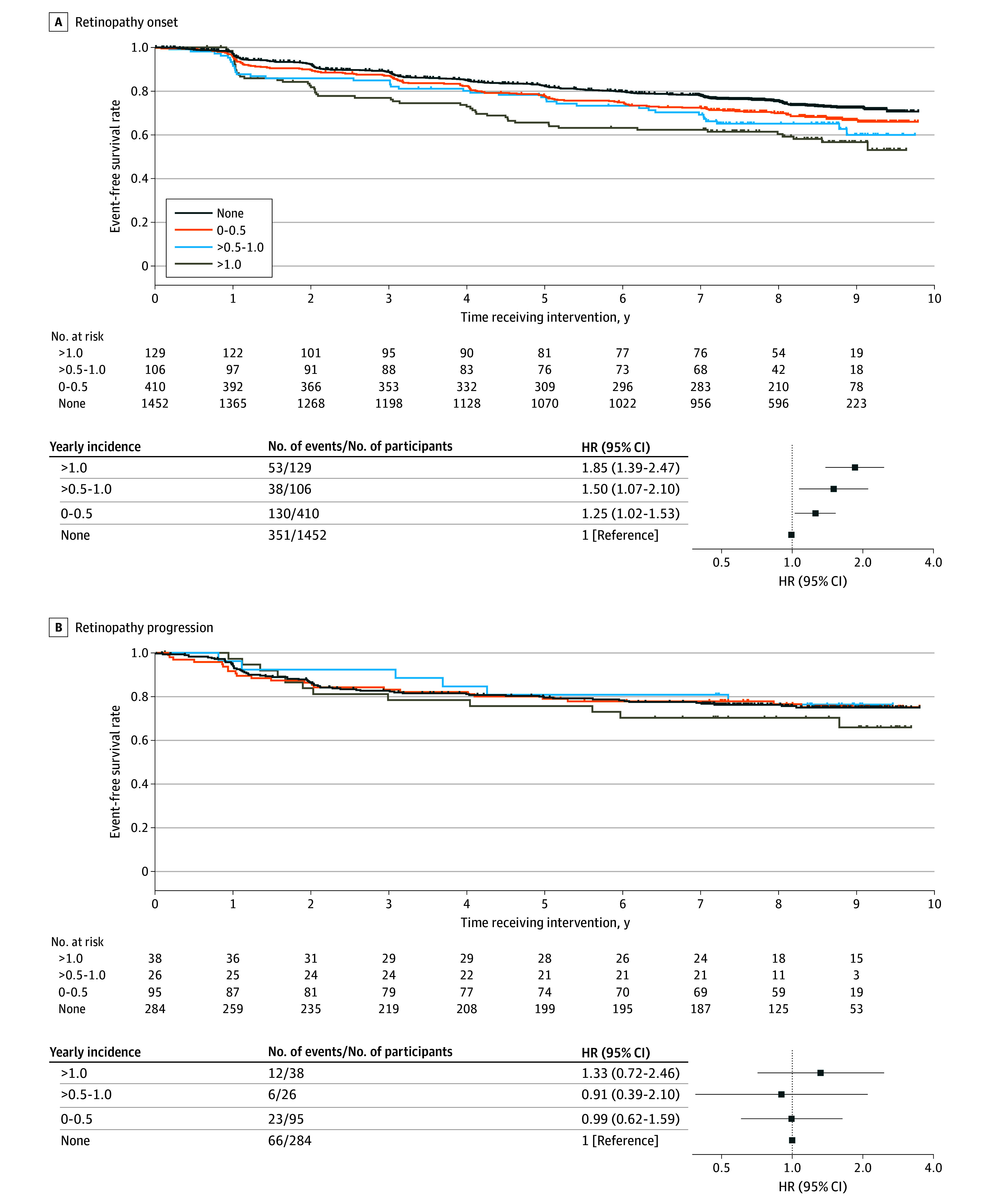
Effects of Hypoglycemia on Retinopathy Events Kaplan-Meier curves and forest plots to show event-free survival rates of onset (A) and progression (B) of retinopathy. Participants were stratified by the presence or absence of retinopathy at baseline and further by the yearly incidence of hypoglycemic episodes during the intervention. In total, 572 onset events were observed in 2097 participants, whereas 107 progression events were observed in 443 participants. Bars represent 95% confidence intervals. HR indicates hazard ratio.

Compared with those without hypoglycemic episodes, the risk of onset was higher in those with 0.5 hypoglycemic episodes per year or fewer (HR, 1.25; 95% CI, 1.02-1.53) and even higher in those with more than 1 episode per year (HR, 1.85; 95% CI, 1.39-2.47) (*P* for trend <.001) (Figure 3A). By contrast, the yearly incidence of hypoglycemia was not significantly associated with progression (Figure 3B). The results were similar, even when participants were further stratified by treatment group (eFigure 1 in Supplement 2) or when we focused on those not receiving a blood glucose meter (eFigure 2 in Supplement 2). These data suggest that avoidance of hypoglycemia could be also associated with prevention of onset of retinopathy.

## Discussion

An intensified multifactorial intervention in the J-DOIT3 trial successfully demonstrated a risk reduction in onset of retinopathy. Moreover, the risk of onset was reduced by better glycemic management but elevated by hypoglycemia.

To the best of our knowledge, this is the first study to examine the effects of not only intensified multifactorial intervention, but also glycemic management and nonsevere hypoglycemia, on onset and progression of retinopathy, respectively, in a clinical trial setting. Good glycemic management (mean HbA_1c_ around 7%) and a low incidence of severe hypoglycemia (<0.1% during 8.5 years), as well as good management of blood pressure and lipids, were all achieved in this trial. Still, the importance of glycemic management is proven, even when most participants (>85%) were treated without insulin,^28^ although the effects of glycemic management have been controversial, especially in type 2 diabetes, in previous trials.^2,3,4,5,6,7,8,13^

The lack of clear HbA_1c_ threshold for retinopathy is consistent with the DCCT study in type 1 diabetes,^2,9^ but not with the Kumamoto study in type 2 diabetes.^4^ It is a strength of this study that we could examine the effect of HbA_1c_ on onset of retinopathy by excluding that of hypoglycemia, because the incidence of hypoglycemia was almost constant, irrespective of HbA_1c_. It should be noted that in the current study, the lower limit of 95% confidence intervals of HR was more than 1.0 for HbA_1c_ of about 7.5% or higher, and the upper limit of HR was close to, but not less than, 1.0 for HbA_1c_ of about 6.0% (Figure 2A).

The association between nonsevere hypoglycemia and onset, not progression, of retinopathy is another novel finding of this study. The risk is elevated by 25% with fewer than 0.5 hypoglycemic episodes per year, an impact almost equivalent to that of an increase in HbA_1c_ by 1%, which elevates the risk by 31%, and risk is almost doubled with more than 1 hypoglycemic episode per year. While clinicians have largely focused attention on the association between hypoglycemia and progression of retinopathy thus far, the effect of hypoglycemia on onset could have been masked by less strict glycemic management in previous studies.^8^ Given the neovascularizing effect of insulin,^37^ inadequately high insulin level observed in hypoglycemia might affect vascular endothelium and potentially trigger onset of retinopathy.

Taken together, it is vitally important to achieve strict and yet safe glycemic management in treating people with type 2 diabetes. Although better glycemic management could be achieved without an elevated risk of hypoglycemia, rapid improvement in glycemic management (eg, a decrease in HbA_1c_ by ≥1% during the first year after initiation of treatment) could elevate the risk of hypoglycemia. Indeed, the incidence of retinopathy events in this study was relatively higher in the first year after randomization (Figure 1A), when medications for diabetes were intensified. Thus, the HbA_1c_ target should be individualized, but glycemic management with an HbA_1c_ target of 7% or lower may be recommended in a certain proportion of people with type 2 diabetes. So-called newer classes of antidiabetic drugs are expected to help clinicians achieve strict and safe glycemic management.^22,38,39^

Among the other risk factors identified in this study are duration of diabetes and comorbid nephropathy, which could account for the lower incidence of retinopathy events (10.2%-12.1% at 5 years) and the absence of statistically significant effect of multifactorial intervention on retinopathy events in the ADDITION-Europe trial, despite a risk reduction (HR, 0.84)^24^ equivalent to that in this study (HR, 0.86). Lower BMI is another risk factor for onset, suggesting that people with type 2 diabetes in East Asia characterized by impaired insulin secretion could gain less weight (and possibly less muscle mass^40^) but may be susceptible to retinopathy.^41^ Higher systolic blood pressure at baseline was another risk factor, but systolic blood pressure at 1 year was not a risk factor, possibly due to the better blood pressure management in this trial.^22,28^

### Limitations

In this trial, the data on photocoagulation, antivascular endothelial cell growth factor treatment, and vitreous surgery were not reported. In addition, regression of retinopathy was not examined as an outcome. Moreover, fundus examination photos and other severity scaling scores were not collected as data. Thus, we could not examine the details or evaluate macular edema as in previous trials,^2,4,5,8,10,11,13,29^ although we clearly identified what was associated with the diagnosis with onset. Lastly, the effects of lipid-lowering with fibrates suggested by recent evidence^5,18,20,21,42^ were difficult to examine. Many participants were treated with a statin (80.9% vs 62.6%), but only a few of them were treated with a fibrate, even in the conventional group (0.6% vs 3.9%),^28^ probably because their combination was contraindicated. It should be examined whether fibrate treatment could improve the benefit of multifactorial intervention in future studies.

## Conclusions

In this secondary analysis of the J-DOIT3 randomized clinical trial, intensified multifactorial intervention achieving good glycemic management and a very low incidence of severe hypoglycemia was associated with a risk reduction in onset of retinopathy in Japanese people with type 2 diabetes. The risk of onset was elevated in parallel with HbA_1c_, even with treatment of hyperglycemia not dependent on insulin therapy, with no clear threshold. Moreover, a hypoglycemic episode was also associated with an increased risk of onset, suggesting the importance of achieving strict glycemic management and preventing hypoglycemia.

## Notes

Supplement 1. Trial Protocol

Supplement 2. eTable 1. Baseline Characteristics eTable 2. The Number of the First Retinopathy Events Stratified by Retinopathy at Baseline eTable 3. Multivariable Cox Regression Analysis of Onset or Progression of Retinopathy With Baseline Characteristics as Covariates eTable 4. Multivariable Cox Regression Analysis of Onset of Retinopathy With Baseline Characteristics as Covariates eTable 5. Multivariable Cox Regression Analysis of Progression of Retinopathy With Baseline Characteristics as Covariates eTable 6. HbA1C Change During the First Year of Intervention and Hypoglycemia eTable 7. Yearly Incidence of Hypoglycemia During the Intervention Period eFigure 1. Effects of Hypoglycemia on Onset of Retinopathy in Both Therapy Groups eFigure 2. Effects of Hypoglycemia on Onset of Retinopathy in Participants Without Receiving a Blood Glucose Meter

Supplement 3. J-DOIT3 Study Group

Supplement 4. Data Sharing Statement
