# Supplementary material for: Effect of a Multifactorial Intervention on Retinopathy in People With Type 2 Diabetes: A Secondary Analysis of the J-DOIT3 Randomized Clinical Trial
Source: JAMA Ophthalmol. 2025 Oct 23;143(12):989–97. doi: 10.1001/jamaophthalmol.2025.3819 (PMC12550739; doi:10.1001/jamaophthalmol.2025.3819)
Supplement: Supplement 1. — Trial Protocol [file jamaophthalmol-e253819-s001.pdf]

J-DOIT3

A randomized controlled study comparing intensive therapy and conventional therapy in the reduction of vascular complications in type 2 diabetic patients

J-DOIT3 Clinical Trial Protocol

A Ministry of Health, Labor and Welfare-sponsored Strategic Outcome Study

Version 1.0 drafted January 26, 2006
Version 1.1 revised January 28, 2006
Version 1.2 revised February 20, 2006
Version 1.3 revised April 19, 2006
Version 1.4 revised June 7, 2006
Version 1.5 revised December 15, 2006
Version 1.6 revised October 1, 2007
Version 1.6.1 revised December 2, 2008
Version 2.0 revised January 25, 2010
Version 2.1 revised January 28, 2011
Version 3.0 revised December 2, 2012
Version 3.1 revised July 8, 2015

Study design and patient assignment: an outline

Trial subjects: Type 2 diabetic patients
Patients aged 45 years or older but younger than 70 years at study entry who satisfy both (1) and (2) or both (1) and (3) or all (1), (2), and (3) below.

(1) Blood glucose level
HbA1c ≥ 6.9% (no medication or 1 oral anti-diabetic agent or 1 oral anti-diabetic agent + α-glucosidase inhibitor [αGI])

(2) Blood pressure control
Blood pressure control ≥ 140/90 mmHg (no medication antihypertensive drug) or ≥ 130/80 mmHg (on ARB, ACE inhibitor or long-acting CCB alone or a combination of them)

(3) Lipid metabolism
LDL-C ≥ 120 mg/dL or TG ≥ 150 mg/dL or HDL-C < 40 mg/dL (no medication or 1 lipid-lowering agent)

Exclusion →

Poorly controlled hypertension
Insulin therapy
Non-diabetic renal disease
Anti-GAD antibody positivity
Suspected type 1 and other diabetes due to pathogenic mechanisms other than type 2 diabetes
LDL-C ≥ 200 mg/dL
Suspected secondary hypertension
Hereditary lipid metabolic abnormalities
Use of antihypertensive drugs other than ARB, ACEI or long-acting CCB
Use of 3 or more antihypertensive drugs including ARB, ACEI or long-acting CCB
More serious retinopathy than proliferative retinopathy
Renal failure (creatinine [Cr], ≥ 2.0 mg/dL in men; ≥ 1.5 mg/dL in women)
History of cardiac failure or its complications
Pregnancy or potential pregnancy
BNP ≥ 100 pg/mL (history of ischemic heart disease [IHD], left ventricular hypertrophy [LVH])
Ineligibility as assessed by physician in charge

↓

Random assignment
Adjusted for presence/absence of history of CVD, sex, age and HbA1c

1669 patients ↓ 1669 patients ↓

Intensive therapy
Management goals:
HbA1c, < 6.2%
Blood pressure control, < 120/75 mmHg
LDL-C, < 80 mg/dL (non-HDL-C, < 100 mg/dL)
HDL-C, ≥ 40 mg/dL; triglycerides (TG), < 120 mg/dL
(LDL-C, < 70 mg/dL [non-HDL-C, < 100 mg/dL] in those with a history of IHD)
(Those with TG ≥ 150 mg/dL to be assessed in terms of non-HDL-C values)
BMI, ≤ 22

Conventional therapy
Management goals:
HbA1c, < 6.9%
Blood pressure control, < 130/80 mmHg
LDL-C, < 120 mg/dL
Triglycerides (TG), < 150 mg/dL
(LDL-C, < 100 mg/dL in those with a history of IHD)
BMI, ≤ 24

Abbreviations: αGI, Alpha-glucosidase inhibitor; ARB, Angiotensin II receptor antagonist; ACEI, Angiotensin-converting enzyme inhibitor; CCB, Calcium channel blocker; CVD, cardiovascular disease (myocardial infarction, stroke, coronary artery bypass graft [CABG], percutaneous coronary angioplasty [PTCA], cerebral revascularization); IHD, ischemic heart disease; LVH, left ventricular hypertrophy

Intensive therapy: an outline of the therapeutic regimens

Lifestyle modification

Target BMI value: ≤ 22; caloric restriction; salt restriction: ≤ 6 g/day

Exercise (15-30-minute walk twice or more daily), alcohol abstinence, smoking cessation

Glycemic control

If each of the steps described below achieves an HbA1c value of less than 6.2% or a 1% reduction in HbA1c values in the subjects within 6 months, that treatment is to be continued; and if not, the treatment is intensified to the next step.

Pioglitazone which is associated with a certain level of evidence that supports their use in the reduction of macrovascular complications are to be chosen whenever possible.

Diet/exercise therapy alone in the initial 3 months
(Subjects on oral anti-diabetic agents are to continue their medications as well)

Step 0

All subjects: Use diet/exercise therapy (+ category D).

Step 1

Subjects with BMI ≥ 25: Give category A agents.
Those with BMI 22 to < 25: Give a category A or B agents.
Those with BMI < 22: Give a category B or A agents.
(+ category D)

Step 2

All subjects: Give category A agents + category B agents (+ category D).

Step 3

Give category C agents (+ category A, B, and D).

Types of therapeutic agents
Category A: pioglitazone, BG, GLP-1 receptor agonists
Category B: SU and glinides
Category C: Insulin
Category D: αGIs, DPP-4 inhibitors, SGLT2 inhibitors

- Anti-D agents within the same drug class (except category B) may be coadministered.
- In category A, pioglitazones which are associated with a certain level of evidence that supports their use in the reduction of macrovascular complications are to be chosen as the agents of first choice.
- Category D agents may be added in any of the steps after paying attention to concomitant drugs.
- Only those agents which are listed in the Package Insert may be coadministered.
- Temporary or continued insulin therapy may be used in any of the steps described above at the discretion of the physician in charge.

Blood pressure control

Blood pressure to be lowered with the goal of achieving < 120/75 mmHg within 3 to 6 months of treatment.

Diet/exercise therapy alone in the initial 3 months
(Subjects on an oral agent are to continue their medications as well)

Step 1

Use an ARB or ACEI with the dose being escalated to its maximal dose.

Step 2

Add a long-acting CCB.

Step 3

Add on a diuretic, β blocker or α blocker, one agent at a time in this order.

Subjects with blood pressure ≥ 160/100 mmHg are to be given oral antihypertensive therapy immediately.

Lipid control

Each of the steps described below is to continue for 3 months. If the goal is achieved in a particular patient with the treatment given, that treatment is to be continued; if not, the treatment is intensified to the next step.
Management goals: LDL-C < 80 (or LDL-C < 70 if history of IHD), TG < 120

Diet/exercise therapy alone in the initial 3 months
(Subjects on oral anti-diabetic agents are to continue their medications, but to discontinue fibrates)

Step 1

Give a standard dose of atorvastatin, pitavastatin or rosuvastatin.

Step 2

Use the agents used in step 1 with the dose escalated to its maximal dose.

Step 3

Add on an anion-exchange resin and/or ezetimibe in any order.

Omega-3 fatty acids are to be given once the goal for LDL-C (non-HDL-C) control has been achieved or if the goal for TG control has not been achieved in step 3.

Abbreviations: αGI: α-glucosidase inhibitors; BG: biguanides; SU: sulfonylureas; ARB, angiotensin receptor blockers; ACEI, angiotensin-converting enzyme inhibitors.

1. Outline of the clinical trial protocol

1.1 Objectives

As an attempt to address one of many research tasks that remain highly unmet in a large Japanese population and thus call for definitive solutions, the present outcome trial represents a large-scale "strategic clinical study" with clearly defined outcome goals, which has been designed to address one of the three major tasks as part of the "Strategic Studies Aimed at the Prophylaxis of Diabetes Mellitus" as defined in the Ministry of Health, Labor and Welfare-designated scientific research project, "Research on Development of Strategic Outcome Studies", in a randomized controlled trial in type 2 diabetic patients to verify the hypothesis that intensive multi-factorial therapy is superior to conventional therapy in preventing the onset and progression of vascular complications associated with diabetes, with the primary endpoint being the occurrence of either of myocardial infarction, coronary bypass surgery, percutaneous transluminal coronary angioplasty, stroke, percutaneous transluminal cerebral angioplasty, carotid endarterectomy, carotid artery stenting, or death.

1.2 Inclusion criteria

Subjects were considered eligible for inclusion if they were 45 years old or older but younger than 70 years old at study entry, had type 2 diabetes and met both "(1) and (2)" or both "(1) and (3)" described below. Those who met all three "(1), (2) and (3)" were also considered eligible for inclusion.

(1) Glycemic control
Those with HbA1c 6.9% or greater despite treatment with any of the three regimens given below.
① Diet and exercise therapy alone
② Diet and exercise therapy plus 1 oral anti-diabetic drug
③ Diet and exercise therapy plus αGI and 1 other oral anti-anti-diabetic drug

(2) Blood pressure control
Those with the following casual blood pressure (BP) level as measured on an outpatient basis
① Systolic BP ≥ 140 mmHg or diastolic BP ≥ 90 mmHg while not on an antihypertensive agent
② Systolic BP ≥ 130 mmHg or diastolic BP ≥ 80 mmHg while on 1 or 2 ARB, ACEI or long-acting CCB
Those receiving antihypertensive agents other than ARB, ACEI or long-acting CCB were not eligible for study entry, with the exception of those who were receiving these agents for other purposes than blood pressure lowering.

(3) Lipid control
Those with the following fasting lipid levels while not on a lipid-lowering agent
① LDL-cholesterol, ≥ 120 mg/dL (as estimated by using the Friedewald formula)
② Triglycerides, ≥ 150 mg/dL
③ HDL-cholesterol, < 40 mg/dL
Subjects receiving 1 lipid-lowering agent were judged eligible for study entry if they met any of the above criteria. However, those on fibrates were to discontinue that fibrate treatment at the start of the study when they were assigned to the intensive therapy arm.

1.3 Exclusion criteria

1. Those with poorly controlled hypertension despite pharmacological therapy (systolic BP ≥ 200 mmHg or diastolic BP ≥ 120 mmHg)
2. Those on insulin therapy
3. Those with non-diabetic renal disease
4. Those in whom type 1 and other diabetes due to pathogenic mechanisms other than those associated with type 2 diabetes is strongly suspected
5. Those who tested anti-GAD antibody-positive
6. Those with LDL-cholesterol ≥ 200 mg/dL
7. Those suspected of having secondary hypertension other than renal parenchymal hypertension
8. Those suspected of having hereditary lipid disorder with a strong family history of lipid metabolic disorder
9. Those who were receiving antihypertensive agents other than ARB, ACEI, long-acting CCB, except where they were receiving these agents for other purposes than blood pressure lowering
10. Those who were receiving 3 or more antihypertensive agents (i.e., ARB, ACEI, and long-acting CCB), except where they were receiving these agents for other purposes than blood pressure lowering
11. Those with more serious retinopathy than proliferative retinopathy
12. Renal failure (serum Cr: ≥ 2.0 mg/dL in men; ≥ 1.5 mg/dL in women)
13. Those with a history of cardiac failure or those with cardiac failure
14. Those who were pregnant or potentially pregnant
15. Those who met any of the following criteria and who had BNP ≥ 100 pg/mL
    - Myocardial infarction
    - Angina pectoris (or a history of disease)
    - History of coronary artery bypass graft (CABG)
    - History of percutaneous coronary angioplasty (PTCA)

- Other cardiac disease
- ECG findings of left ventricular hyperplasia
- Abnormal ECG findings (excluding isolated extrasystole or right bundle branch block [RBBB])

16. Those judged by the attending physician to be ineligible for study entry

1.4 Investigational treatment

Subjects judged eligible for the study were randomly assigned to intensive multi-factorial therapy or conventional therapy. The management goals set for either therapy were described below.

Management goals for intensive therapy and conventional therapy

	Management goals	
	Intensive therapy	Conventional therapy
Lifestyle modification		
Weight reduction	BMI, ≤ 22	BMI, ≤ 24
Diet restriction	Total energy intake to be rigorously controlled (25 kcal/kg in those with BMI ≥ 25; 27 kcal/kg in those with BMI < 25) with lipid intake accounting for less than 25% of total energy intake, cholesterol intake accounting for 300 mg/day or less, and salt intake accounting for 6 g/day or less; alcohol and tobacco abstinence to be rigorously adhered to	Appropriate diet therapy in accordance with the Japan Diabetes Society Clinical Practice Guidelines
Exercise therapy	2 or more 15- to 30-minute walks on a daily basis	Appropriate exercise therapy in accordance with the Japan Diabetes Society Clinical Practice Guidelines
Glycemic control	HbA1c, < 6.2%	HbA1c, < 6.9%
Blood pressure control	Systolic BP, < 120 mmHg *and*<br>Diastolic BP, < 75 mmHg	Systolic BP, < 130 mmHg *and*<br>Diastolic BP, < 80 mmHg
Lipid control	LDL-C, < 80 mg/dL[*1]<br>TG, < 120 mg/dL<br>HDL-C, ≥ 40 mg/dL<br>Except in those with a history of coronary artery disease (CAD), where the goal is to be set at LDL-C < 70 mg/dL[*1]	LDL-C, < 120 mg/dL<br>TG, < 150 mg/dL<br><br>Except in those with a history of coronary artery disease (CAD), where the goal is set at LDL-C < 100 mg/dL.

[*1] In those with TG ≥ 150 mg/dL, the subjects are to be assessed in terms of non-HDL-C (= total cholesterol – HDL-C) levels, with the management goal set as non-HDL-C < 110 mg/dL, except in those with a history of CAD, where it is set as non-HDL-C < 100 mg/dL.

1.5 Physical and laboratory examinations during planned patient visits

Physical and clinical examinations during planned patient visits include:
Body weight, blood pressure control;
Blood glucose, HbA1c (only stable HbA1c to be measured for adjustment against reference material);
Total cholesterol, LDL-cholesterol (non-HDL-cholesterol), HDL-cholesterol, triglycerides;
Hematologic test (white blood cell count, red blood cell count, hemoglobin, hematocrit, platelet count), hepatic/renal function test (AST, ALT, γ-GPT, LDH, BUN, serum Cr), serum electrolytes (Na, K, Cl), CPK; and
Urinary albumin, and urinary Cr (to be measured every 6 months).

1.6 Primary endpoint of the study

The primary endpoint of the study is defined as the occurrence of either of myocardial infarction, coronary bypass surgery, percutaneous transluminal coronary angioplasty, stroke, carotid endarterectomy, percutaneous transluminal cerebral angioplasty, carotid artery stenting, or death (irrespective of its causes).

1.7 Secondary endpoints of the study

The secondary endpoints of the study include:
① Occurrence of either of myocardial infarction, stroke or death
② Onset or progression of nephropathy
③ Lower limb vascular events (amputation or revascularization of lower limb)
④ Onset or progression of retinopathy

1.8 The trial duration and number of participating healthcare institutions

The study is to remain open for patient accrual for a period of 2.75 years until March 2009, with the subjects being followed up until the number of events for the primary endpoint reaches 250 (expected duration from participation to March 2016).

1.9 Target patient accrual

The target patient accrual is projected at 1,669 each for intensive and conventional therapy, totaling 3,338 patients. Note: The actual number of subjects totaled 2,542 patients, or 1,271 patients each for intensive and conventional therapy.

2. Table of contents

1. Outline of the clinical trial protocol 5
   1.1 Objectives 5
   1.2 Inclusion criteria 5
   1.3 Exclusion criteria 5
   1.4 Investigational treatment 6
   1.5 Physical and laboratory examinations during planned patient visits 6
   1.6 Primary endpoint of the study 6
   1.7 Secondary endpoints of the study 6
   1.8 The trial duration and number of participating healthcare institutions 6
   1.9 Target patient accrual 7
2. Table of contents 8
3. List of abbreviations used in this document 11
4. Background 12
5. Objectives of the study 13
6. Study design 13
   6.1 Processes 13
   6.2 Target patient accrual and trial duration 14
   6.3 Rationale behind the study design 14
7. Criteria for patient inclusion, exclusion and treatment discontinuation 15
   7.1 Inclusion criteria 15
   7.2 Exclusion criteria 15
   7.3 Criteria for treatment discontinuation in individual subjects 16
8. Investigational treatment 17
   8.1 Guidance on lifestyle modification 17
      8.1.1 Weight reduction 17
      8.1.2 Diet restriction 17
      8.1.3 Exercise therapy 17
      8.1.4 Smoking cessation 17
      8.1.5 Implementation of the core program 17
   8.2 Glycemic control 18
      8.2.1 Intensive therapy 18
      8.2.2 Conventional therapy 19
      8.2.3 Rationale for the goal setting 19
   8.3 Blood pressure control 20
      8.3.1 Intensive therapy 20
      8.3.2 Conventional therapy 21
      8.3.3 Rationale for the goal setting 21
   8.4 Lipid control 21

8.4.1 Intensive therapy 21
8.4.2 Conventional therapy 22
8.4.3 Rationale for the goal setting 22
8.5 Other treatments 22
8.6 Random assignment 22
9. Observation/evaluation schedule 23
9.1 Items to be examined at informed consent (from the time of informed consent until before interim 24
9.2 Items to be examined at final study registration 24
9.3 Items to be examined at regular hospital visits 24
9.4 Items to be examined every 6 months 24
9.5 Items to be examined every 12 months 24
9.6 Clinical parameters to be examined centrally 25
9.7 Other surveys 25
9.8 Reporting the Data Center 25
9.9 Feedback from the Data Center 25
9.10 General investigations 25
10. Efficacy evaluation 26
10.1 Primary endpoint 26
10.2 Secondary endpoints 26
10.3 Definition of the endpoints used 26
10.4 Assessment of the endpoints 26
10.5 Reporting on events 26
11. Safety evaluation 27
11.1 Technical terminology 27
11.2 Collection of data on adverse reactions 27
11.3 Severity of adverse reactions and their causal relationship to the investigational treatment 28
11.4 Serious adverse reactions 28
11.4.1 Technical terminology 28
11.4.2 Reporting procedure 28
11.4.3 Assessment by the Clinical Research Leader and the Trial Secretariat Office 28
11.5 Analysis of adverse reactions through regular monitoring 29
11.6 Adverse reactions to be anticipated 29
12. Statistical analysis 29
12.1 Objective 29
12.2 Subjects of analysis 29
12.3 Statistical methods 30
12.3.1 Primary analyses 30
12.3.2 Secondary analyses 30
12.3.3 Other analyses 30

12.4 Interim Analyses and the Clinical Trial Review Committee 31
12.4.1 Interim analyses 31
12.4.2 Reporting on/review of statistical analyses 31
12.4.3 Follow-up in the event of early trial termination 32
12.4.4 Final analyses 32
13. Target patient accrual and the rationale for the target setting 32
14. Ethical considerations 32
14.1 Observance of the ethical criteria 32
14.2 Ethics Review Committees 32
14.3 Patient informed consent 33
14.4 Protection of privacy and identification of the study subjects 34
15. Quality management 34
15.1 Procedures for case report development and patient data consolidation 34
15.2 Data monitoring 34
15.3 Auditing 34
16. Data handling and archiving 34
17. Policy for data disclosure 34
18. Violations and revisions of the trial protocol 34
18.1 Violation of the protocol to avoid emergencies and crises 34
18.2 Revision of the trial protocol and early termination of the trial 34
19. Trial investigators and committees 35
19.1 Principal Investigator 35
19.2 Clinical Research Leader 35
19.3 Trial Coordinating Committee 35
19.4 Trial Administrative Committee 35
19.5 Trial Assessment Committee 35
19.6 Trial Progress Management 36
19.7 Safety Assessment Committee 36
19.8 Central Ethics Committee 36
20. Research funding 36
21. Conflicts of interest 37

3. List of abbreviations used in this document

Abbreviation	Expansion
AST	Aspartate aminotransferase (used as a synonym for glutamic-osaloacetic transaminase [GOT])
ALT	Alanine aminotransferase (used as a synonym for glutamic-pyruvic transaminase [GPT])
BDI	Beck Depression Inventory
BMI	Body mass index
BNP	B-type natriuretic peptide
BUN	Blood urea nitrogen
CPK	Creatine phophokinase
CRP	C-reactive protein
DBP	Diastolic blood pressure
DHQ	Dietary History Questionnaire
GAD	Glutamic acid decarboxylase
GFR	Glomerular filtration rate
γ-GTP	γ-glutamyltanspeptitase
HbA1c	Hemoglobin A1c (glycosylated hemoglobin)
HDL-C	High-density lipoprotein cholesterol
LDH	Lactate dehydrogenase
LDL-C	Low-density lipoprotein cholesterol
Non-HDL-C	Non-high-density lipoprotein cholesterol
PAID	Problem Area in Diabetes Survey
PHRF-SCL (SF)	Public Health Research Foundation Stress Check List (Short Form)
PROactive	Prospective pioglitazone clinical trial in macrovascular events
SBP	Systolic blood pressure
SF-36	Medical Outcomes Study-Short-Form 36-Item Health Survey
UKPDS	United Kingdom Prospective Diabetes Study

4. Background

Diabetes mellitus constitutes a group of metabolic diseases primarily characterized by a chronic hyperglycemic state associated with insufficient insulin action which leads to disorders in glycemic, lipid control and protein metabolism. Long-lasting metabolic disorders lead to diabetic complications, namely vascular complications becoming manifest over time. Commonly, macrovascular lesions involving large vessels, such as coronary artery, cerebral artery, and artery of the lower extremities, account for myocardial infarction, cerebral infarct, and neuropathy in the lower extremities, whereas microvascular lesions account for visual impairment, renal failure, and neuropathy. Epidemiological studies including the Hisayama Study demonstrate that diabetes is associated with a close to 3-fold risk for the onset of macrovascular disease such as myocardial infarction and stroke, and this risk becomes still greater when it is complicated by hypertension or dyslipidemia. Furthermore, a total of 13,000 patients are estimated to start receiving artificial dialysis annually due to nephropathy as a microvascular disease, with 3,500 patients estimated to suffer a loss of sight annually due to retinopathy.

Japanese patients with diabetes represent a sizable population, accounting for some 16.2 million people, including those strongly suspected of having diabetes totaling some 7.4 million and those in whom diabetes could not be ruled out as a possibility, and, of these, those receiving treatment for the disease account for only 3.7 million, according to a fact-finding study conducted in 2002 by the Ministry of Health, Labor and Welfare. Furthermore, the study indicated that even those receiving treatment were not necessarily being managed properly, with only 9 hundred thousand patients being maintained at appropriate glucose levels (hemoglobin A1c [HbA1c], < 6.9%), and the remaining 2.8 million individuals being maintained at suboptimal glucose levels. This situation points to the need for establishing an appropriate management approach to diabetes that may help prevent the onset of vascular complications.

Globally, a number of clinical studies evaluating various anti-diabetic therapeutic strategies have been conducted to provide evidence that aggressive and intensive therapy produces positive outcomes. Of these, the United Kingdom Prospective Diabetes Study 33 (UKPDS 33) in which a total of 3,867 type 2 diabetic patients were randomly assigned to intensive therapy with sulfonylurea (SU)/insulin or to conventional diet therapy for glycemic control is noteworthy in that it demonstrated that intensive therapy leads to a decrease in the risk among the treated patients for onset of microvascular complications, compared to diet therapy alone (Lancet 1998;352:837-853). Furthermore, in the UKPDS 35 in which the results of the UKPDS were further analyzed epidemiologically, it was shown that decreases in HbA1c go hand in hand with decreases in the risk for mortality, myocardial infarction, and microvascular complications (BMJ 2000;321:405-412). Again, the Steno-2 Study in 100 type 2 diabetic patients with microalbuminurea randomly assigned to intensive therapy for rigorous glycemic, blood pressure and lipid control or to conventional therapy reported a reduction in the incidence of vascular complications in patients treated with composite intensive therapy (N Engl J Med 2003;348:383-93). More recently, the PROactive study (PROspective pioglitAzone Clinical Trial In macroVascular Events) demonstrated in 5,238 patients with type 2 diabetes randomly assigned to pioglitazone or to placebo in addition to conventional therapy that pioglitazone added to conventional therapy produced greater reductions in the onset of macrovascular complications than placebo, indicating that intensive therapy is efficacious in secondary prophylaxis of macrovascular complications (Lancet 2005;366:1279-89).

However, no studies have been performed in Japan, to date, to evaluate the role of therapeutic intervention in the reduction of macrovascular complications, while there were a number of studies conducted to evaluate the role of intervention in the reduction of microvascular complications, and therefore, it still remains unclear how effective intensive therapy may be in reducing macrovascular complications of diabetes. Against this background, the current study set out to evaluate, as its primary endpoint, whether or not intensive therapy might produce greater reductions in the onset of macrovascular complications than conventional therapy by randomly assigning the subjects to intensive therapy aimed at rigorous lifestyle, glycemic, blood pressure and lipid control or to conventional therapy aimed at treating patients to current standards. Furthermore, given that patients being started on dialysis due to diabetic nephropathy account for as many as 13,000 annually, and that the prophylaxis of onset or progression of nephropathy has great social and economic consequences, the present study also evaluated the onset and progression of nephropathy as a secondary endpoint.

The management goals for either treatment arm in this study were determined in light of the Japan Diabetes Society (JDS) Evidence-based Clinical Practice Guidelines for the Management of Diabetes (hereafter Guidelines) as well as overseas clinical trial results reported to date. Specifically, the goals for conventional therapy were decided on in light of the Guidelines, while those for intensive therapy, more rigorous than those for conventional therapy, were determined in light of available overseas clinical trial data.

Of note, the subjects in the present study are highly unlikely to be associated with a greater risk than those being treated in the clinical setting, given that they are to be treated with approved anti-diabetic, antihypertensive and anti-dyslipidemic drugs at the doses approved for clinical use. Therefore, the risk associated with their random assignment should remain within acceptable range. Conversely, the benefit associated with their participation in the current study is unlikely to be greater than being treated in the clinical setting. However, if the current study provides evidence that supports the superiority of intensive therapy over conventional therapy, it should prove helpful in developing future therapeutic strategies for diabetes; in other words, it should promise to offer benefits to Japanese type 2 diabetic patients at large.

5. Objectives of the study

The present study is designed to verify in a randomized controlled trial in type 2 diabetic patients the hypothesis that intensive therapy is superior to conventional therapy in the prophylaxis of onset or progression of vascular complications associated with diabetes, with the primary endpoint being the "occurrence of either of myocardial infarction, coronary bypass surgery, percutaneous transluminal coronary angioplasty, stroke, carotid endarterectomy, percutaneous transluminal cerebral angioplasty, carotid artery stenting, or death" and the secondary endpoints being the "occurrence of either of myocardial infarction, stroke or death", "onset or progression of nephropathy", "onset of lower limb vascular event" (amputation or revascularization of lower limb), and "onset or progression of retinopathy".

6. Study design

6.1 Processes

The present trial is a multi-center, open-label, randomized parallel-group study conducted to compare the efficacy of intensive versus conventional therapy in type 2 diabetic patients with HbA1c 6.9% or greater. The overall procedure from patient informed consent to completion of patient data evaluation is as described below.

(1) Informed consent

In obtaining informed consent, the physician in charge is to explain to each candidate subject in person who is considered to meet all inclusion criteria and not to meet any exclusion criteria to obtain the subject's consent to participate in the study in writing, which, however, must be obtained one or more days after the date of physician explanation. The informed consent obtained in writing is to be photocopied in duplicate, one copy for the subject himself/herself and the other for the host healthcare institution, with the original copy kept on file in the subject's medical record.

(2) Interim study registration

After obtaining informed consent, the physician in charge is to perform examinations on items set forth in section 9.1 in each subject. However, available chest x ray, electrocardiogram (ECG) and funduscopic data for the subject that is up to 6 months old may alternatively be used. The physician in charge is to forward his judgment as to the eligibility of the subject to the central Data Center for the subject to be enrolled in the study on an interim basis.

Based on the information forwarded by the physician in charge, the Data Center is to verify the eligibility of the subject thus registered, and to inform the physician in charge of the subject's interim registration when the subject was found eligible. The Data Center is also to inform the physician in charge of its judgment when the subject was found otherwise (ineligible).

(3) Final study registration

The physician in charge is to perform examinations on items set forth in section 9.2 in all subjects judged eligible at interim registration, and to assign and register them.

Final study registration is to be completed one day after but within 3 months of the date of informed consent.

The Data Center is to randomly assign all subjects to intensive therapy or to conventional therapy, and to inform the physician in charge that they have been registered with the study and their allocation numbers assigned. Random assignment is to be adjusted by the presence or absence of cardiovascular disease (myocardial infarction or stroke, coronary bypass surgery, percutaneous coronary angioplasty, cerebral revascularization[*1], or cerebrovascular reconstruction[*2]), male/female ratio, age and HbA1c to ensure a balanced allocation of the subjects between the treatment arms (i.e., dynamic allocation with the presence or absence cardiovascular disease, male/female ratio, age and HbA1c serving as adjustment factors).

In the event that the Data Center judged a particular subject ineligible for final study registration for some reason, the Data Center is to inform the physician in charge of its decision and rationale behind the decision.

The physician in charge is to perform examinations on items set forth in section 9.2 in all subjects at their final registration.

Note: Cerebral revascularization[*1]: Carotid endarterectomy, percutaneous angioplasty, and stent placement
Cerebrovascular reconstruction[2*]: Thrombolysis

(4) Investigational treatment and monitoring of events

The physician in charge is to provide all subjects allocated by the Data Center with guidance on lifestyle modification, treat them for glycemic, blood pressure and lipid control, and to monitor for macrovascular and microvascular complications and death from completion of their study registration until the number of events for the primary endpoint reaches 250.

Table 1. Management goals for intensive therapy and conventional therapy

	Management goals	
	Intensive therapy	Conventional therapy
Lifestyle modification		
Weight reduction	BMI, ≤ 22	BMI, ≤ 24
Diet restriction	Total energy intake to be rigorously controlled (25 kcal/kg in those with BMI ≥ 25; 27 kcal/kg in those with BMI < 25) with lipid intake accounting for less than 25% of total energy intake, cholesterol intake accounting for 300 mg/day or less, and salt intake accounting for 6 g/day or less; alcohol and tobacco abstinence to be rigorously adhered to	Appropriate diet therapy in accordance with the Japan Diabetes Society Clinical Practice Guidelines
Exercise therapy	2 or more 15- to 30-minute walks on a daily basis	Appropriate exercise therapy in accordance with the Japan Diabetes Society Clinical Practice Guidelines
Glycemic control	HbA1c, < 6.2%	HbA1c, < 6.9%
Blood pressure control	Systolic BP, < 120 mmHg *and* Diastolic BP, < 75 mmHg	Systolic BP, < 130 mmHg *and* Diastolic BP, < 80 mmHg
Lipid control	LDL-C, < 80 mg/dL[*1]<br>TG, < 120 mg/dL<br>HDL-C, ≥ 40 mg/dL<br>Except in those with a history of coronary artery disease (CAD), where the goal is to be set at LDL-C < 70 mg/dL[*1]	LDL-C, < 120 mg/dL<br>TG, < 150 mg/dL<br>Except in those with a history of coronary artery disease (CAD), where the goal is set at LDL-C < 100 mg/dL.

[*1] In those with TG ≥ 150 mg/dL, the subjects are to be assessed in terms of non-HDL-C (= total cholesterol – HDL-C) levels, with the management goal set as non-HDL-C < 110 mg/dL, except in those with a history of CAD, where it is set as non-HDL-C < 100 mg/dL.

6.2 Target patient accrual and trial duration

The target patient accrual is projected at 1,669 each for intensive and conventional therapy, totaling 3,338 patients. The study is to remain open for patient accrual for a period of 2.75 years until March 2009, with the subjects being followed up for 4 years from their entry until the number of events for the primary endpoint reaches 250, involving a total of 81 healthcare institutions that each allows 50 or more subjects (25 or more subjects per group) to be accrued. The actual number of subjects totalled 2,542 patients, or 1,271 patients each for intensive and conventional therapy.

6.3 Rationale behind the study design

As a randomized controlled trial in type 2 diabetic patients with HbA1c 6.9% or greater, the present study sets out to verify the hypothesis that intensive therapy significantly reduces the onset or progression of vascular complications associated with diabetes. The reason that the study involves type 2 diabetic patients is that they represent a population at high risk of developing fatal and non-fatal macrovascular complications. The criterion of HbA1c 6.9% or greater was determined based on the Guidelines. Given that the Guidelines define HbA1c 6.9% as "inadequate glycemic control" and as a category requiring lowering HbA1c to less than 6.9%, the inclusion criterion for type 2 diabetic patients with inadequate glycemic control was defined in the present study as "HbA1c 6.9% or greater".

The management goals for the study were also determined in light of the Guidelines, where HbA1c 6.2% or less is defined as "excellently controlled", and HbA1c less than 6.9% as "well controlled", so that HbA1c 6.2 was defined as the management goal for intensive therapy, and HbA1c less than 6.9% defined as the management goal for conventional therapy. As no categories are given for blood pressure and lipid control such as "excellently controlled" or "well controlled", the management goals given in the Guidelines were chosen for conventional therapy, while the management goals for intensive therapy were determined in light of overseas clinical trial data reported to date. In assigning the subjects to the two treatment arms, dynamic allocation was used with important prognostic factors serving as adjustment factors to ensure balanced allocation between the arms.

The present study is designed to verify that the event-free survival period (time to occurrence of either of myocardial infarction, coronary bypass surgery, percutaneous transluminal coronary angioplasty, stroke, carotid endarterectomy, percutaneous transluminal cerebral angioplasty, carotid artery stenting, or death) of patients who receive intensive therapy is longer than that of patients who receive conventional therapy, with the primary endpoint being the "occurrence of either of myocardial infarction, coronary bypass surgery, percutaneous transluminal coronary angioplasty, stroke, carotid endarterectomy, percutaneous transluminal cerebral angioplasty, carotid artery stenting, or death" and the secondary endpoints being the "occurrence of either of myocardial infarction, stroke or death", "onset or progression of nephropathy", "onset of lower limb vascular event" (amputation or revascularization of lower limb), and "onset or progression of retinopathy". Therefore, the current study is intended to evaluate the role of intensive versus conventional therapy both in the primary prophylaxis of these events (i.e., prevention of their onset in those without a history of these

events) and in their secondary prevention (i.e., prevention of their recurrence or progression in those with a history of these events). The sample size for either arm in the current study was determined based on the assumption that patients with a history of these events and those without account for 30% and 70% of the entire target population, respectively.

7. Criteria for patient inclusion, exclusion and treatment discontinuation

7.1 Inclusion criteria

Subjects were considered eligible if they were 45 years old or older but younger than 70 years old at study entry, had type 2 diabetes and met both "(1) and (2)" or both "(1) and (3)" described below. [Note: Those who met all three "(1), (2) and (3)" were also considered eligible for inclusion.]

(1) Glycemic control
Those with HbA1c 6.9% or greater despite treatment with any of the three regimens given below.
① Diet and exercise therapy alone
② Diet and exercise therapy plus 1 oral anti-diabetic drug
③ Diet and exercise therapy plus αGI and 1 other oral anti-anti-diabetic drug

(2) Blood pressure control
Those with the following casual blood pressure (BP) level as measured on an outpatient basis
① Systolic BP ≥ 140 mmHg or diastolic BP ≥ 90 mmHg while not on an antihypertensive agent
② Systolic BP ≥ 130 mmHg or diastolic BP ≥ 80 mmHg while on 1 or 2 ARB, ACEI or long-acting CCB
Those receiving antihypertensive agents other than ARB, ACEI or long-acting CCB were not eligible for study entry, with the exception of those who were receiving these agents for other purposes than blood pressure lowering.

(3) Lipid control
Those with the following fasting lipid levels while not on a lipid-lowering agent
① LDL-cholesterol, ≥ 120 mg/dL (as calculated by using the Friedewald formula)
② Triglycerides, ≥ 150 mg/dL
③ HDL-cholesterol, < 40 mg/dL
Subjects receiving 1 lipid-lowering agent were judged eligible for study entry if they met any of the above criteria. However, care needs to be taken to ensure that those on fibrates discontinue the fibrate treatment at the start of the study when they are assigned to the intensive therapy arm.

7.2 Exclusion criteria

Those who meet any of the following are to be excluded from the study, despite their having met the inclusion criteria.
1. Those with poorly controlled hypertension despite pharmacological therapy (systolic BP ≥ 200 mmHg or diastolic BP ≥ 120 mmHg)
2. Those on insulin therapy
3. Those with non-diabetic renal disease
4. Those in whom type 1 and other diabetes due to pathogenic mechanisms other than those associated with type 2 diabetes is strongly suspected
5. Those who tested anti-GAD antibody*-positive
6. Those with LDL-cholesterol ≥ 200 mg/dL
7. Those suspected of having secondary hypertension other than renal parenchymal hypertension
8. Those suspected of having hereditary lipid disorder with a strong family history of lipid metabolic disorder
9. Those who were receiving antihypertensive agents other than ARB, ACEI, long-acting CCB, except where they were receiving these agents for other purposes than blood pressure lowering
10. Those who were receiving 3 or more antihypertensive agents (i.e., ARB, ACEI, and long-acting CCB), except where they were receiving these agents for other purposes than blood pressure lowering
11. Those with more serious retinopathy than proliferative retinopathy
12. Renal failure (serum Cr: ≥ 2.0 mg/dL in men; ≥ 1.5 mg/dL in women)
13. Those with a history of cardiac failure or those with cardiac failure
14. Those who were pregnant or potentially pregnant
15. Those who met any of the following criteria and who had BNP ≥ 100 pg/mL
- Myocardial infarction
- Angina pectoris (or a history of disease)
- History of coronary artery bypass graft (CABG)
- History of percutaneous coronary angioplasty (PTCA)
- Other cardiac disease
- ECG findings of left ventricular hyperplasia
- Abnormal ECG findings (excluding isolated extrasystole or right bundle branch block [RBBB])

16. Those judged by the physician in charge to be ineligible for study entry

[Rationale for these criteria]
1: Due to its potential interference with efficacy evaluation and associated safety concerns

2-10: Due to potential interference with efficacy evaluation
11-16: Due to associated safety concerns
*Anti-GAD antibody: an autoantibody against the islet-derived molecule glutamic acid decarboxylase (GAD), which is known to be useful in the differential diagnosis of type 2 diabetes

7.3 Criteria for treatment discontinuation in individual subjects

If the investigational treatment cannot be continued in individual subjects for reasons listed below, the physician in charge is to discontinue the treatment in these subjects, record the date of discontinuance along with the reasons for discontinuance, and report to the Data Center.

- Occurrence of a serious macrovascular or microvascular disease
- Occurrence of one other serious complicating disease
- Diagnosis or strong suspicion of type 1 diabetes (including slowly progressive IDDM; SPIDDM)
- Difficulties continuing treatment due to adverse reactions
- Patient request for discontinuance
- Patient death
- Other cases in which the physician in charge judged continued treatment difficult

Even when the investigational treatment was discontinued in a particular subject, with the subject's consent (for subjects who are incompetent, consent from the family member or legally authorized representative), the subject can be followed up on the presence or absence of events set forth in section 10 as the primary and secondary endpoints of the study. In which case, in accordance with the observation/evaluation schedule laid down in section 9, serum creatinine, urinary albumin and urinary creatinine are to be measured, and funduscopic and ECG exams to be performed in the subject.

If any subject ceases to present at the host hospital, the physician in charge is to inquire the subject by phone or mail as to his/her reasons for not presenting and to confirm his/her willingness to continue to participate in the study (if the subject is able to resume his/her regular visits, encourage him/her to do so). If, however, the subject is judged to be having difficulty continuing the investigational treatment, the physician in charge is to determine whether or not the subject had any of the events described in section 10. If the subject was found to be dead, the physician in charge is to look into the date, cause and location (home address) of death of the subject. As soon as the physician in charge found it difficult to make any further survey on the subject, he/she is to make entries in the medical record about when, how and why the subject discontinued the investigational treatment, as well as if the subject had any of the events set forth in section 10 at the time of treatment discontinuation, and report to the Data Center.

If the subject was found to have moved out or changed hospital, the physician in charge is to identify the location of the subject as well as the hospital through appropriate means, and inquire the subject as to his/her willingness to continue with the investigational treatment, and determine whether or not the subject had any event, in much the same way as the physician in charge is to deal with a subject ceasing to present at the hospital. As soon as the physician in charge found it difficult to make any further survey on the subject, he/she is to make entries in the medical record about when, how and why the subject discontinued the investigational treatment, as well as whether or not the subject had any event at the time of treatment discontinuation, and report to the Data Center.

If the physician in charge had any difficulty obtaining information about the subject from the new hospital, the Data Center is to consider intervening to get necessary information as required. In which case, however, the Data Center is to ask that the physician in charge at the new hospital obtain consent from the subject (for subjects who are incompetent, consent from the family member or legally authorized representative) himself/herself regarding release of the information being sought. Alternatively, the Data Center is to contact the subject (for subjects who are incompetent, the family member or legally authorized representative) directly to obtain such information.

If the Data Center had any difficulty following up on the subject, the Data Center is to consider sending a Clinical Research Leader-designated agent to find out whether or not the subject is dead or alive where he/she lives by consulting the Basic Residence Register. Additionally, if permission is obtained to do so from the Ministry of Internal Affairs and Communications, the Data Center is to consider sending a Clinical Research Leader-designated agent to look into the cause of death of the subject by consulting the Vital Statistics. The processes through which to implement these inquiries, along with that that concerns protection of the subject's privacy, are described elsewhere.

8. Investigational treatment

The investigational treatment in this study is defined as "intensive multi-factorial therapy" with "conventional therapy" serving as the reference/control treatment, where each treatment is intended to achieve lifestyle modification (weight reduction, diet restriction, exercise therapy and smoking cessation) as well as glycemic, blood pressure and lipid control as described below. In using pharmacologic agents for glycemic, blood pressure and lipid control, it is essential that relevant package inserts be consulted to ensure that they are not used in patients in whom their use is contraindicated.

8.1 Guidance on lifestyle modification

8.1.1 Weight reduction

All subjects in both treatment arms are to be provided with patient diaries to record their own body weight.

In the intensive therapy arm, where the management goal for BMI is set for 22 or less, all subjects are obliged to measure their body weight at a predetermined time once daily, record and report on the measurement, and the physician in charge is to keep informed of these measurements.

In the conventional therapy arm, where the management goal for BMI is set for 24 or less, all subjects are also obliged, as are those in the investigational treatment arm, to measure their body weight, record and report on the measurement, and the physician in charge is to keep informed of these measurements.

Once the management goal for BMI (body weight) has been achieved in any patients in either arm, BMI is to be maintained at that level. In those complicating diabetic nephropathy, the management goal is to be achieved by drawing on the diet criteria for diabetic nephropathy.

Between-meal and bedtime snacks are to be prohibited as a rule, except where they are required to avoid hypoglycemia associated with the use of oral anti-diabetic drugs or insulin therapy.

8.1.2 Diet restriction

Total energy intake is to be rigorously controlled in the subjects in the intensive therapy arm. Specifically, in those with BMI 25 or greater at study entry, the management goal for diet restriction is set for "25 kcal/1 kg of ideal body weight (IBW)", and in those with BMI less than 25, the goal for diet restriction is set for "27 kcal/1 kg of ideal body weight (IBW)".

In this regard, the subjects in the intensive therapy arm are to be instructed in person that through diet restriction, lipid is to account for less than 25% of total energy intake, with cholesterol intake accounting for 300 mg/day or less and salt intake accounting for 6 g/day or less. Additionally, they are to be instructed that between-meal and bedtime snacks are to be prohibited, and alcohol abstinence (equivalent to 180 mL of sake or less) is to be adhered to, and they are obliged to report on alcohol intake at regular hospital visits.

Additionally, they are to be given 30-minute or longer guidance on nutrition by the designated national registered dietitians at the start of the study, 1, 3, 6 and 12 months after the start of the study and every 6 months in the second year afterwards (a separate manual on nutritional guidance is to be developed to ensure consistency in their approach).

The subjects in the conventional therapy arm are to be instructed about the total energy intake to be adhered to in accordance with the Japan Diabetes Society Clinical Practice Guidelines (hereafter Guidelines). Salt restriction is also to be encouraged as required.

Subjects in both arms are to be instructed on allowable diets using the "Food Exchange Lists - Dietary Guidance for Persons with Diabetes (7th edition)" edited by the Japan Diabetes Society. Again, those with diabetic nephropathy in both arms are to be encouraged to restrict protein intake in accordance with the "A Guide to the Management of Diabetes" edited by the Japan Diabetes Society.

8.1.3 Exercise therapy

All subjects in both treatment arms are to be loaned an accelerometer at study entry.

The subjects in the intensive therapy arm are to be instructed to walk 15 to 30 minutes twice or more frequently per day. As a rule, the subjects are obliged to walk every day and to report on the calorie consumed as well as the number of steps taken every day. The physicians or nurses in charge are to keep informed of their activity status so that it can be borne in mind when giving exercise instructions.

The subjects in the conventional therapy arm are to be instructed in accordance with the Guidelines.

8.1.4 Smoking cessation

All subjects in the intensive therapy arm are to be instructed to persevere in their smoking cessation practice and to report on the number of cigarettes smoked at their regular hospital visits. Those who find smoking cessation difficult are to be encouraged to use smoking-cessation aids.

8.1.5 Implementation of the core program

To ensure that the goals for lifestyle modification (weight reduction, dietary restriction, exercise and smoking cessation) are met in the intensive therapy arm, a core program intended to improve lifestyle habits is to be implemented to ensure lifestyle factors are improved in the subjects. A post-core program will also be implemented after completion of the core program to ensure lifestyle factors are further improved in the subjects. Details of the core program are given elsewhere.

8.2 Glycemic control

8.2.1 Intensive therapy

In the intensive therapy arm, the goal for glycemic control is defined as HbA1c < 6.2% and the subjects are to make regular hospital visits for glycemic control and monitoring (see Note 1). If any of the subjects receiving treatment on an outpatient basis does not achieve the management goals, they will be immediately hospitalized to ensure better glycemic control in these subjects.

Furthermore, all subjects in the intensive therapy arm are to be loaned a blood glucose meter for self-monitoring of blood glucose (SMBG), and the physicians in charge are to keep informed of the measured results in these subjects.

All subjects are to be treated first with predetermined diet and exercise therapy, and if they fail to achieve HbA1c < 6.2% in the first 3 months, they will be given anti-diabetic drugs in the following steps (those already on anti-diabetic drugs at study entry are to continue with the medication and be treated with lifestyle modification to achieve their respective management goals). Anti-diabetic drugs to be used include thiazolidinedione derivatives, biguanides, sulfonylureas, rapid-onset insulin secretagogues (glinides), α-glucosidase inhibitors, DPP-4 inhibitors, SGLT2 inhibitors, GLP-1 receptor antagonists and insulin. Of these, sulfonylureas and glinides are both designated as "insulin secretagogues" and DPP-4 inhibitors and GLP-1 receptor agonists are designated as "incretin-related drugs."

The treatment specified in each of the steps is to be continued if the treatment leads to HbA1c < 6.2% or a ≥ 1% decrease in HbA1c within 6 months for the subjects. If, however, the goal of HbA1c < 6.2% cannot be achieved with treatment and if treatment is associated with a less than 1% decrease in HbA1c, the treatment is to be intensified to the next step (refer to Notes 1 and 2 if only one of the goals is achieved). In any of the steps described, the dosage may be increased, decreased, discontinued or changed (see Note 2), and an α-glucosidase inhibitor, DPP-4 inhibitor, or SGLT2 inhibitor may be added at the discretion of the physician in charge. For instructions on the addition of a biguanide or GLP-1 receptor agonist, see Note 3 and, for directions for use of incretin-related drugs, see Note 4.

The thiazolidinedione derivative pioglitazone is to be given up to 30 mg/day as a standard maximal dose but the dose can be further increased to 45 mg/day if the physician in charge finds it necessary. However, utmost care is to be taken in increasing the dose from 30 mg to 45 mg/day.

Again, if the physician in charge finds it necessary, temporary or continued insulin therapy can be given (this constitutes step 3). In this case, however, a thiazolidinedione derivative is to be used in combination as long as that is possible. Whether or not to use a thiazolidinedione in combination is to be decided at the discretion of the physician in charge.

In any of the steps described below, diet and exercise therapy is to be continued.

Step 0:
If the goal of obtaining an HbA1c < 6.2% is achieved with diet and exercise therapy plus or minus an α-glucosidase inhibitor, DPP-4 inhibitor and/or SGLT2 inhibitor within the first 3 months, that particular treatment is to be continued (step 0).

Step 1:
The subjects are to be treated with a drug from among those listed below according to their BMI values at study entry. However, thiazolidinedione derivatives associated with a certain level of evidence that supports their use are to be used whenever possible. If any of the subjects are already receiving the drug at study entry, the treatment is intensified to step 2.

① A thiazolidinedione derivative (or possibly a biguanide or GLP-1 receptor agonist) in those with BMI ≥ 25
② A thiazolidinedione derivative (or possibly a biguanide or GLP-1 receptor agonist) or an insulin secretagogue in those with 22 ≤ BMI < 25
③ An insulin secretagogue or thiazolidinedione derivative (or possibly a biguanide or GLP-1 receptor agonist) in those with BMI < 22

Step 2:
Depending on the drug used at study entry or in step 1, an appropriate choice (① or ②) is to be made.
① Add an insulin secretagogue if a thiazolidinedione derivative (biguanide or GLP-1 receptor agonist) is already being used
② Add a thiazolidinedione (or possibly a biguanide or GLP-1 receptor agonist) if an insulin secretagogue is already being used

Step 3:
Insulin therapy is to be initiated. The insulin regimen may be chosen from among the following, and also changed during the course of treatment, in accordance with the patient's symptoms:
① Intermediate- or long-acting insulin injection once daily before bedtime
② Intermediate- or mixed insulin injection twice daily (before breakfast and dinner)
③ Rapid- or ultra-rapid-onset injection insulin three times daily (before meals)
④ Rapid- or ultra-rapid-onset insulin injection three times daily (before meals) plus intermediate- or long-acting insulin injection once daily before bedtime

All subjects are to be given a thiazolidinedione derivative whenever possible even after they have moved on to insulin therapy. The physician in charge is to be responsible for any decision made on the use of an anti-diabetic drug other than a thiazolidinedione derivative in combination with insulin therapy.

Note 1: The frequency of hospital visits will be at least once a month until March 2010 and, thereafter, as set forth below.
① As a rule, at least once a month in those patients receiving insulin therapy and those patients with unstable glycemic

control.
② Maximum of 3 months apart in those patients who have almost achieved management goals for glycemic control and other factors and whose condition is stable (except patients receiving insulin therapy).
Note 2: The physician in charge may at any time replace one insulin secretagogue with another at his/her discretion as required, i.e., replacing a sulfonylurea with a glinide or vice versa. Additionally, the physician in charge may at any time switch among a thiazolidinedione, biguanide, or GLP-1 receptor agonist or use a combination thereof, at his discretion. Also, the physician in charge may at any time replace one drug with another within the same drug class.
Note 3: When a biguanide or GLP-1 receptor agonist has been added, the treatment is intensified to the next step, except in the following cases:

- When a biguanide or GLP-1 receptor agonist was given in subjects with HbA1c < 6.2%
- When a biguanide or GLP-1 receptor agonist was given in subjects receiving a thiazolidinedione derivative

When adequate glycemic control has not been achieved with a biguanide combined with a GLP-1 receptor agonist and/or an insulin secretagogue, a thiazolidinedione derivative is to be given whenever possible. In which case, where a thiazolidinedione derivative was given to subjects already receiving a biguanide or a GLP-1 receptor, the duration of therapy for step 2 is to be 6 months from the time when the thiazolidinedione derivative was started.
Note 4: When an incretin-related drug is used in combination with an SU, etc., the drugs should be administered in accordance with the respective Package Insert for each drug while paying careful attention to the occurrence of hypoglycemia. Moreover, incretin-related drugs should be administered in accordance with the Recommendations on Appropriate Use of Incretin-Related Drugs.
Note 5: If an SGLT2 inhibitor is administered, the drug should be used in accordance with the Package Insert for the drug while paying careful attention to the onset of dehydration or other symptoms. If an SGLT2 inhibitor is used in combination with insulin or an SU, etc., the drugs should be administered in accordance with the respective Package Inserts for each drug while paying careful attention to the onset of hypoglycemia. Moreover, SGLT2 inhibitors should be administered in accordance with the Recommendations on Appropriate Use of SGLT2 Inhibitors.

Dose reduction and discontinuation of anti-diabetic drugs
The physician in charge is to carefully watch for episodes of hypoglycemia in all subjects receiving anti-diabetic drugs, and to adequately educate the subjects on how to deal with these episodes when they appear. In the subjects with frequent hypoglycemia, every effort is to be made to immediately optimize drug therapy by having them consult the physician in charge as often as possible or by hospitalizing them. In optimizing drug therapy, the decision as to which drug needs to be discontinued or reduced is to be left up to the physician in charge. However, even when the physician in charge makes such a decision, he/she is to avoid discontinuing the thiazolidinedione derivative already being used whenever possible. When fluid retention occurs likely due to the use of a thiazolidinedione derivative, the physician in charge is to deal with it by giving a diuretic or by reducing or discontinuing the thiazolidinedione derivative at his discretion.

Note 1: If the goal of HbA1c < 6.2% has been achieved with drug therapy, that particular treatment is to be continued. However, the decision is left up to the physician in charge on whether or not to increase, reduce or discontinue a particular drug, replace a sulfonylurea with a glinide or vice versa, switch treatment among a thiazolidinedione derivative, biguanide, or GLP-1 receptor agonist, replace a drug with another within the same drug class, or add an α-glucosidase inhibitor, DPP-4 inhibitor, SGLT2 inhibitor, biguanide, or GLP-1 receptor agonist. When HbA1c becomes 6.2% or greater in a particular subject, every effort is to be made to ensure that HbA1c is maintained below 6.2% by implementing more intensive therapy (more rigorous lifestyle modification, dose increases or replacements). In the event that HbA1c becomes 6.2% or greater on 3 consecutive measurements despite this intensive therapy in a particular subject, it is to be deemed difficult to achieve the predetermined management goals in this patient, the treatment is intensified to the next step depending on the management goals achieved in this patient as follows:
Diet/exercise therapy (+α-glucosidase inhibitor, DPP-4 inhibitor and/or SGLT2 inhibitor) (considered as step 0) → step 1
1 anti-diabetic drug (+α-glucosidase inhibitor, DPP-4 inhibitor and/or SGLT2 inhibitor) (considered as step 1) → step 2
2 anti-diabetic drugs (+α-glucosidase inhibitor, DPP-4 inhibitor and/or SGLT2 inhibitor) (considered as step 2) → step 3
Note 2: If HbA1c has decreased by more than 1% in a particular patient in the first 6 months, the same treatment in the treatment step is to be continued to ensure that HbA1c is maintained below 6.2% or decreases by more than 1% in the next 6 months.
Note 3: When a particular patient was started on insulin therapy, this is to be construed as constituting step 3. If that patient stops requiring insulin therapy at a later date, the patient is to be construed as being stepped down to that which is consistent with the drug therapy he/she happens to be receiving at that time.

8.2.2 Conventional therapy
The goal for glycemic control in the conventional therapy arm is defined as HbA1c < 6.9%, and the physician in charge is to administer appropriate therapy in accordance with the Guidelines.

8.2.3 Rationale for the goal setting
The goal for glycemic control in the intensive therapy arm was defined as HbA1c < 6.2%, based on the upper limit of normal in individuals with normoglycemia, which the Guidelines defines as "excellently controlled".
The goal for glycemic control in the conventional therapy arm was defined as HbA1c < 6.9%, in light of the threshold value defined for prevention of onset or progression of microvascular complications, which the Guidelines defines as "well controlled".

8.3 Blood pressure control

All the subjects in both treatment arms are to be loaned a blood pressure manometer at study entry.

8.3.1 Intensive therapy

The goal for blood pressure control in the intensive therapy arm is defined as "systolic blood pressure < 120 mmHg and diastolic blood pressure < 75 mmHg" as measured on an outpatient basis.

All subjects in the intensive therapy arm are obliged to measure their blood pressure control by using the loaned blood pressure manometer, record and report on the measurement, and the physician in charge is to keep informed of these measurements as a basis for decisions regarding antihypertensive drug dose escalation or replacement. All subjects are to be treated first with predetermined diet and exercise therapy (those already on antihypertensive drugs at study entry are to continue with the medication to achieve their management goal), and if they fail to achieve the blood pressure goal in the first 3 months, they will be given antihypertensive drugs in the following steps to ensure that the blood pressure goal is achieved in 3 to 6 months. However, in those with systolic blood pressure 160 mmHg or greater or diastolic blood pressure 100 mmHg or greater, diet and exercise therapy, where blood pressure control is to be only monitored, is to be skipped, and antihypertensive therapy is to be immediately initiated by following the steps shown below.

In any of the steps described below, diet and exercise therapy is to be continued.

Step 1:
Give an angiotensin receptor blocker (ARB) or angiotensin-converting enzyme inhibitor (ACEI) at its standard dose with the dose being escalated to its maximal dose as required until the blood pressure goal is achieved in a particular subject. If the goal cannot be achieved even with the maximal dose of the ARB or ACEI, the treatment is intensified to step 2.
Step 2:
Add a long-acting calcium channel blocker (CCB) to the antihypertensive drug used, with the dose being escalated as required. Those already receiving a long-acting CCB, the treatment is intensified to step 3.
Step 3:
Add on a diuretic, β-blocker or α-blocker, one agent at a time as required, in this order.

Drug dose reduction or discontinuation is to be decided at the discretion of the physician in charge, where the decision as to which drug is to be discontinued or by how much the dose needs to be reduced is to be left up to the physician in charge. The physician in charge may at any time replace an ARB with an ACEI or the other way around, or replace one drug with another within the same drug class at his/her discretion.

In those receiving 1 long-acting CCB at study entry and in whom the blood pressure goal cannot be achieved within the first 3 months, the long-acting CCB is to be switched to an ARB or ACEI (step 1). Again, if the goal for blood pressure control could not be achieved in these subjects even when an ARB or ACEI was added to the long-acting CCB with its dose escalated to its maximal dose, they are to be moved up to step 3. In this regard, the physician in charge may decide in which step a particular subject is to be treated.

Blood pressure control after achievement of the goal for blood pressure control
If desired blood pressure control is maintained with drug therapy in a particular patient beyond the period specified above, that particular treatment is to be continued, irrespective of which step the patient happens to be in (although the physician in charge may reduce the drug dose or discontinue the drug at his/her discretion).

If blood pressure control begins to exceed the blood pressure goal in this patient at a later date, the patient is to be treated as shown below.

If the patient is in step 1 or 2, the drug therapy designated in that treatment step is to be used with the drug dose being increased or another drug added or with enhanced diet and exercise therapy, where blood pressure control in excess of the blood pressure goal as confirmed on 3 consecutive measurements is to be construed as an indication for the next step up.

If the patient is in step 3, the patient is to be treated to ensure that he/she achieves the goal for blood pressure control, if possible, by increasing the dose of the drug he/she is receiving, adding another drug or enhancing the diet and exercise therapy being given.

Note: If a particular patient called for no blood pressure control at study entry and already achieved the blood pressure goal, he/she is to be treated in accordance with the treatment step in which he/she is being treated. However, if the patient called for no blood pressure control at study entry but has not achieved the blood pressure goal, he/she is to be treated consistently with the treatment step in which he/she is being treated and observed for 3 months. If the patient has achieved the blood pressure goal at the end of the observation period, the same treatment is to be continued; however, once his/her blood pressure begins to exceed the blood pressure goal, he/she is to be treated as shown below:

Those on no antihypertensive drugs are to be moved to step 1 to receive a standard dose of an angiotensin receptor blocker (ARB) or angiotensin-converting enzyme inhibitor (ACEI).
Those on an ARB or ACEI alone are to be moved to step 1 and treated with the dose being escalated as required to its maximal dose (Those on 2 ARB/ACEI or more are to be treated with the dose of one of these agents being escalated to its maximal dose).
Those on a long-acting CCB and ARB or ACEI are to be treated with the dose of the ARB or ACEI being escalated to its

maximal dose, and to be moved up to step 3 if the treatment failed.
Those on a long acting CCB alone are to be switched to an ARB or ACEI and moved up to step 1. Alternatively, they are to be given a standard dose of an ARB or ACEI, with the dose being escalated as required to its maximal dose, and then to be moved up to step 3 if the treatment failed. The physician in charge may choose between these two options at his/her discretion.

8.3.2 Conventional therapy

The goal for blood pressure control in the conventional therapy arm was defined as "systolic blood pressure < 130 mmHg and diastolic blood pressure < 80 mmHg" as measured on an outpatient basis. The physicians in charge are to choose appropriate therapy for the subjects in the conventional therapy arm in accordance with the Guidelines.

8.3.3 Rationale for the goal setting

The goal for blood pressure control in the intensive therapy arm was defined as "systolic blood pressure < 120 mmHg and diastolic blood pressure < 75 mmHg", in light of the available overseas large-scale randomized controlled trial data. In the United Kingdom Prospective Diabetes Study (UKPDS) 36 conducted in 1,148 patients whose blood pressure control was tightly controlled, an analysis of the relationship between the incidence of diabetic complications and systolic blood pressure in these subjects, as adjusted for age, gender and race, suggested that a monotonic decrease occurs in the incidence of diabetic complications in proportion to the blood pressure control lowered in those with systolic blood pressure ranging between 115 mmHg to 170 mmHg (BMJ 2000;321:412-9.). Furthermore, a meta-analysis of 61 prospective trials evaluating the impact of blood pressure control on cardiovascular mortality demonstrated that in all age groups 40 years old or older (40s, 50s, 60s and 70s), blood pressuring lowering is associated with a monotonic decrease in cardiovascular mortality (Lancet 2002;360:1903-13.). The meta-analysis also demonstrated that the blood pressure goal of systolic blood pressure < 120 mmHg/diastolic blood pressure < 75 mmHg is associated with the least risk for cardiovascular death, which provided a basis for the goal setting for blood pressure control in the intensive therapy arm.
The goal for blood pressure control in the conventional therapy arm was defined as "systolic blood pressure < 130 mmHg and diastolic blood pressure < 80 mmHg", in accordance with the Guidelines.

8.4 Lipid control

8.4.1 Intensive therapy

The goal for lipid control in the intensive therapy arm is defined as "LDL-cholesterol (LDL-C) < 80 mg/dL and triglycerides (TG) < 120 mg/dL and HDL-cholesterol (HDL-C) ≥ 40 mg/dL". However, for those with a history of coronary artery disease (CAD), the goal for LDL-C control is defined as < 70 mg/dL. In those with TG 150 mg/dL or greater, lipid control is to be evaluated in terms of non-HDL-C (= total cholesterol – HDL-C) values, instead of LDL-C values. In which case, the goal for non-HDL-C control is defined as the goal for LDL-C control plus 30 mg/dL. In other words, the goal for non-HDL-C control is defined as < 110 mg/dL in those with no history of CAD and 100 mg/dL in those with a history of CAD.
All subjects are to be treated first with predetermined diet and exercise therapy (those already on lipid-lowering drugs at study entry are to continue with the medication, except for fibrates that need to be discontinued, to achieve their management goal). If they fail to achieve the lipid control goal in the first 3 months, they will be given lipid-lowering drugs in the following steps.
In any of the steps described below, diet and exercise therapy is to be continued.

Step 1:
Give a standard dose of atorvastatin, pitavastatin or rosuvastatin to all subjects who have not achieved the goal for LDL-C (non-HDL-C) control or that for TG control.
Step 2:
The drug given in step 1 is to be used with the dose being escalated as required to the upper limit of the approved dosage.
Step 3:
Add on an anion-exchange resin and/or omega-3 fatty acids in any order.

Omega-3 fatty acids are to be given if the goal for TG control (< 120 mg/dL) has not been achieved after the goal for LDL-C (non-HDL-C) goal has been achieved in step 1 or 2 or if the goal for TG control has not been achieved in step 3.

In all treatment steps, attention is to be focused on improving hypo-HDL-cholesterolemia with exercise, smoking cessation or a thiazolidinedione derivative when it occurs.

Drug dose reduction or discontinuation is to be decided at the discretion of the physician in charge, where the decision as to which drug to discontinue or by how much the dose needs to be reduced is to be left up to the physician in charge. The physician in charge may at any time replace atorvastatin with pitavastatin or rosuvastatin, replace pitavastatin with atorvastatin or rosuvastatin, or replace rosuvastatin with atorvastatin or pitavastatin at his/her discretion.

Lipid control after achievement of the goal
If desired lipid control is maintained with drug therapy in a particular patient beyond the period specified above, that particular treatment is to be continued (although the physician in charge may reduce the drug dose or discontinue the drug at his/her discretion).

If lipid control levels begin to exceed their respective goals in this patient at a later date, the patient is to be treated as shown below.

If the patient is in step 1 or 2, the drug therapy designated in that treatment step is to be used with the drug dose being increased or another drug (omega-3 fatty acids) added or with enhanced diet and exercise therapy, where lipid control (LDL-C [non-HDL-C] and TG) levels in excess of their respective goals as confirmed on 3 consecutive measurements is to be construed as an indication for the next step up.

If the patient is in step 3, attention is to be focused on ensuring that he/she achieves the goal for lipid control, if possible, by increasing the dose of the drug he/she is receiving or enhancing the diet and exercise therapy being given.

Note: In the subjects who were receiving fibrates at study entry and were then assigned to the intensive therapy arm, all fibrates are to be discontinued. If a particular patient called for no lipid control at study entry and already achieved the lipid control goal, he/she is to be treated in accordance with the treatment step in which he/she is being treated. However, if the patient called for no lipid control at study entry but has not achieved the goal for lipid control, he/she is to be treated consistently with the treatment step in which he/she is being treated and observed for 3 months. If the patient has achieved the goal for lipid control at the end of the observation period, the same treatment is to be continued; however, once his/her lipid levels begin to exceed the goal for lipid control, all medications for dyslipidemia are to be discontinued and atorvastatin, pitavastatin or rosuvastin is to be started in the patient. Drug dose is to be determined at the discretion of the physician in charge, and the patient is to be treated in step 1 or 2 depending on the dose, with the subsequent treatment being organized in accordance with the treatment steps for lipid control.

8.4.2 Conventional therapy

The goal for lipid control in the conventional therapy arm is defined as "LDL-cholesterol (LDL-C) < 120 mg/dL and triglycerides (TG) < 150 mg/dL". However, for those with a history of coronary artery disease (CAD), the goal for LDL-C control is defined as < 100 mg/dL. The physicians in charge are to choose appropriate therapy for the subjects in the conventional therapy arm in accordance with the Guidelines.

8.4.3 Rationale for the goal setting

The goal for lipid control in the intensive therapy arm was defined in light of the available overseas large-scale randomized controlled trial data and the analyses of these trial data. Analyses of the effect of lipid-lowering therapy on LDL-cholesterol values and on primary prevention of CAD in randomized controlled trials in patients with hyperlipidemia showed that a monotonic decrease occurs in the incidence of CAD in proportion to the LDL-cholesterol level lowered in those with LDL-cholesterol levels ranging between 90 to 210 mg/dL (Atherosclerosis 1999;143(Suppl. 1):S17-S21; Lancet 2003;361:1149-58). Similarly, an analysis of the effect of lipid-lowering therapy on secondary prevention of CAD showed that a monotonic decrease occurs in the incidence of CAD in proportion to the LDL-cholesterol level lowered in those with LDL-cholesterol levels ranging between 50 to 190 mg/dL (J Am Coll Cardiol 2004;43:2142-6). Furthermore, it was reported in the Collaborative Atorvastatin Diabetes Study (CARDS) conducted in type 2 diabetic patients that improving lipid metabolism leads to a reduction in the incidence of cardiovascular events (Lancet 2004;364:685-96). In setting the goal for lipid control in those with hypertriglyceridemia, non-HDL-cholesterol values were used to assess lipid control, given that the LDL-cholesterol level cannot be accurately estimated in these patients by using the Friedewald formula and that the influence of arteriosclerotic lipoproteins is not negligible in these patients, in addition to LDL-cholesterol.

The goal for lipid control in the conventional therapy arm was defined as "LDL-cholesterol (LDL-C) < 120 mg/dL and triglycerides (TG) < 150 mg/dL" in those with no history of coronary artery disease, and "LDL < 100 mg/dL" in those with a history of coronary artery disease.

8.5 Other treatments

In both the intensive therapy and conventional therapy arms, all subjects with a history of cardiovascular disease are to be given anti-platelet and anti-coagulant therapy such as low-dose aspirin (enteric tablets) in accordance with the Guidelines. All subjects who are already receiving such therapy are to be given the same treatment in both the intensive therapy and conventional therapy arms.

The physician in charge may at his/her discretion use any drugs other than those defined in 8.2, 8.3 and 8.4 for glycemic, blood pressure and lipid control.

8.6 Random assignment

The study subjects are to be assigned to the investigational treatment in which the responsibility for such assignment is to rest solely with the trial statistician, and the allocation code used is not to be disclosed to any of the healthcare institutions or physicians participating in the study. In random assignment, dynamic allocation is used with the presence or absence of cardiovascular disease (myocardial infarction, stroke, coronary bypass surgery, percutaneous coronary angioplasty, cerebral revascularization*1, or cerebrovascular reconstruction*2), male/female ratio, age (≥ 60, < 60 years old) and HbA1c (≥ 8.9%, < 8.9%) serving as adjustment factors to ensure a balanced allocation of the subjects between the treatment arms, and the trial statistician is to record the algorithm used in allocation in a separate document.

Note: Cerebral revascularization[*1]: Carotid endarterectomy, percutaneous angioplasty, and stent placement
Cerebrovascular reconstruction[2*]: Thrombolysis

9. Observation/evaluation schedule

An outline of the observation/evaluation schedule for the study is shown in Table 2.

Table 2. Observation/evaluation schedule: an outline[*1]

	At informed consent[*2]	At final study registration	At regular hospital visits[*3]	Every 6 months	Every 12 months
Height		○			○
Waist circumference		○			○
Body weight		○	○	○	○
Blood pressure control	○	○	○	○	○
Glucose level, HbA1c[*4]	○	○	○	○	○
Total cholesterol, LDL-C[*5] (non-HDL-C[*6]), HDL-C, TG	○	○	○	○	○
Hematologic examinations[*7], hepatic/renal function test[*8], CPK, serum electrolytes (Na, K[*9], Cl)	○	○	○	○	○
Urinary albumin[*10], urinary creatinine (to be measured in the same sample)		○	(○)[*11]	○	○
Brain natriuretic peptide (BNP)	(○) [*12]				
Chest x ray, ECG, fundus examination	○				○
Parameters to be examined centrally[*13]		○			○

[*1] Hematologic examinations scheduled at the time of informed consent (from the time of consent obtained until before interim study registration), at official study entry and every 12 months are to be performed in fasting states.

[*2] Examinations scheduled at the time of informed consent are to be performed from the date consent obtained until before interim study registration.

Items to be examined during this time include: blood pressure control, total cholesterol, LDL-cholesterol (non-HDL-cholesterol), HDL-cholesterol, triglycerides

Chest x ray, echocardiogram (ECG), fundus examination (by an ophthalmologist)

(Available data on these examinations that is up to 6 months old may alternatively be used)

Hepatic/renal function test (serum creatinine), BNP (only in those suspected of meeting the relevant exclusion criteria)

[*3] These are to be examined on a regular basis during the entire study period in conjunction with the hospital visits planned for each subject.

[*4] Only stable HbA1c is to be measured and adjusted against reference material.

[*5] To be estimated by using the Friedewald formula (LDL-C = total cholesterol − HDL-C − TG $\times$ 1/5), except in those with TG levels $\geq$ 400 mg/dL.

[*6] To be estimated by using the formula: non-HDL-C = total cholesterol − HDL-C

[*7] This includes white blood cell count, red blood cell count, hemoglobin, hematocrit, and platelet count.

[*8] This includes AST, ALT, γ-GPT, LDH, BUN, and serum creatinine.

[*9] Those in whom the potassium level ranged between 5.6 and 5.9 mEq/L are to be re-examined. Those in whom the potassium level exceeded 6.0 mEq/L are to be re-examined and treated appropriately.

[*10] Urinary albumin is to be evaluated for amount of its excretion per 1 g of urinary creatinine, and to be evaluated in morning urine samples at final study registration and every 12 months.

[*11] Those who had an episode of nephropathy are to be re-examined within 3 months of its occurrence using a morning urine sample.

[*12] To be performed only in those suspected of meeting the relevant exclusion criteria (including myocardial infarction, angina or a history of angina, a history of coronary bypass surgery, a history of percutaneous coronary angioplasty, other cardiac disease, ECG findings of left ventricular hyperplasia, and abnormal ECG findings)

[*13] Items to be examined and the timeline for these examinations are described in section 9.6.

Examination dates

- Examinations scheduled for implementation at patient hospital visits every 6 months and every 12 months (except

chest X-ray, ECG and fundus exams) are to be performed between 1 month before the predetermined date and 1 month after that date.

- Available chest x ray, ECG and fundus exams that are up to 6 months old may alternatively be used. These examinations scheduled every 12 months are to be performed between 2 months before the predetermined date and 2 months after that date.
- Final study registration is to be completed one day after but within 3 months of the date of informed consent.

9.1 Items to be examined at informed consent (from the time of informed consent until before interim study registration)

The physician in charge is to perform the following examinations after informed consent was obtained from the subjects but before their final study registration. Hematologic examinations are to be performed in fasting states.

Blood pressure control
HbA1c, total cholesterol, LDL-cholesterol (non-HDL-cholesterol), HDL-cholesterol, triglycerides
Chest x ray, echocardiogram (ECG), fundus examination
(Data from these exams that are 6 months old or more recent may alternatively be used).
Hepatic/renal function test (serum creatinine), BNP (only in those suspected of meeting the relevant exclusion criteria)

9.2 Items to be examined at final study registration

The physician in charge is to perform the following examinations at the subjects' final study registration. Hematologic examinations are to be performed in fasting states.

Height, body weight, blood pressure, waist circumference
Fasting plasma glucose, HbA1c, total cholesterol, LDL-cholesterol (non-HDL-cholesterol), HDL-cholesterol, and triglycerides
Hematologic examination (white blood cell count, red blood cell count, hemoglobin, hematocrit, and platelet count), hapetic/renal function test (AST, ALT, γ-GTP, LDH, BUN, and serum creatinine), serum electrolytes (Na, K, and Cl), CPK, urinary albumin (to be measured in a morning urine sample), urinary creatinine (to be measured in the same sample used for urinary albumin measurement), family history, maximum body weight in the past, and disease duration

9.3 Items to be examined at regular hospital visits

The subjects are to be evaluated for the following parameters at their regular hospital visits. Hematologic examinations may be performed in non-fasting states.

Body weight, blood pressure control
Blood glucose, HbA1c, total cholesterol, LDL-cholesterol (non-HDL-cholesterol), HDL-cholesterol, and triglycerides
Hematologic examination (white blood cell count, red blood cell count, hemoglobin, hematocrit, and platelet count), hapetic/renal function test (AST, ALT, γ-GTP, LDH, BUN, and serum creatinine), serum electrolytes (Na, K, and Cl), and CPK

9.4 Items to be examined every 6 months

The subjects are to be evaluated for the following parameters every 6 months. Hematologic examinations may be performed in non-fasting states.

Body weight, blood pressure control
Blood glucose, HbA1c, total cholesterol, LDL-cholesterol (non-HDL-cholesterol), HDL-cholesterol, and triglycerides
Hematologic examination (white blood cell count, red blood cell count, hemoglobin, hematocrit, and platelet count), hapetic/renal function test (AST, ALT, γ-GTP, LDH, BUN, and serum creatinine), serum electrolytes (Na, K, and Cl), and CPK
Urinary albumin, urinary creatinine (to be measured in the same sample used for urinary albumin measurement)

9.5 Items to be examined every 12 months

The subjects are to be evaluated for the following parameters every 6 months. Hematologic examinations are to be performed in fasting states.

Height, body weight, blood pressure control, waist circumference
Fasting plasma glucose, HbA1c, total cholesterol, LDL-cholesterol (non-HDL-cholesterol), HDL-cholesterol, and triglycerides
Chest x ray, echocardiogram (ECG), fundus examination
(Data from these exams obtained within 2 months before or after the predetermined examination date may alternatively be used. The fundus examination is also to be performed within 6 months prior to the end of the study.).
Hematologic examination (white blood cell count, red blood cell count, hemoglobin, hematocrit, and platelet count), hapetic/renal function test (AST, ALT, γ-GTP, LDH, BUN, and serum creatinine), serum electrolytes (Na, K, and Cl), CPK, urinary albumin (to be measured in a morning urine sample), urinary creatinine (to be measured in the same sample used for urinary albumin measurement)

9.6 Clinical parameters to be examined centrally

High-sensitive C-reactive protein (hs-CRP), insulin, adiponectin, and glycoalbumin (GA) are to be examined centrally, using the same samples as for routine measurements. High-sensitive CRP, insulin and GA are to be evaluated at the time of final study registration and every 12 months while adinopectin will be evaluated at the time of final study registration, in March 2013, and at study completion. Residual samples are to be sent to the designated laboratory (SRL) by the Principal Investigator in accordance with the "Ethical Guidelines for Medical and Health Research Involving Human Subjects" and are to be retained appropriately in accordance with the standard operating procedures (SOP).

9.7 Other surveys

Questionnaire-based surveys are to be conducted in subjects prior to final study registration[*1] as well as after 1 and 3 years and at study completion. Subjects who discontinue treatment will be surveyed at the time of discontinuation. In addition, a cognitive function test will be performed at the end of the study. The details of these surveys to be conducted are described in a separate document.

If an assessment of risk factors or frequency of events, other complications, and adverse drug reactions is required, additional parameters will be measured at the central laboratory using residual samples or clinical information will be collected using questionnaires or other tools.

Note:[*1] To be performed after informed consent was obtained from each subject but prior to his/her final study registration.

9.8 Reporting the Data Center

If a macrovascular or microvascular complication occurred in a particular subject, its finding/diagnosis, date of its occurrence, and its causal relationship are to be reported to the Data Center.

To facilitate evaluation of the predetermined endpoints, when myocardial infarction or a coronary event occurred in a particular subject, a photocopy of the echocardiogram (ECG) chart used as a basis for the subject's eligibility evaluation as well as of the ECG chart at the time of the event occurrence is to be submitted to the Data Center. These copies are to be submitted in such a way that the subject can in no way be identified by the Data Center.

9.9 Feedback from the Data Center

The Data Center is to make inquiries to the participating healthcare institutions as to body weight, HbA1c, blood pressure control, and lipid control profile in the subjects assigned to the intensive therapy arm every 6 months to evaluate if they have achieved the management goals for these parameters, and encourage the institutions to ensure these goals are achieved.

9.10 General investigations

During the course of the study, general investigations are to be performed on a regular basis to keep track of the incidence of cardiovascular events as well as to see if the study is in progress exactly as planned.

These general investigations are intended to survey on the occurrence of cardiovascular events during designated time periods. Items to be surveyed include diagnosed events, date of their occurrence, date of examination for event evaluation and event details; or if there were no events to be reported, an indication to that effect as well as the date of final confirmation.

10. Efficacy evaluation

10.1 Primary endpoint

The primary endpoint of the study is defined as the "occurrence of either of myocardial infarction, coronary bypass surgery, percutaneous transluminal coronary angioplasty, stroke, carotid endarterectomy, percutaneous transluminal cerebral angioplasty, carotid artery stenting, or death (irrespective of its causes)".

10.2 Secondary endpoints

The secondary endpoints of the study are defined as the following four events.

1. Occurrence of either of myocardial infarction, stroke, or death
2. Onset or progression of nephropathy
3. Occurrence of lower limb vascular events (amputation or revascularization of lower limb)
4. Onset or progression of retinopathy

10.3 Definition of the endpoints used

A) Myocardial infarction

Myocardial infarction is defined as the presence of typical symptoms (e.g., lasting severe chest pain), echocardiographic changes, abnormal laboratory findings (e.g., elevation of the cardiac enzyme troponin T), or findings of clinical imaging (e.g., coronary angiography, cardiac scintigraphy, multi-detector row computed tomography) which were diagnosed as such by a physician.

B) Stroke

Stroke is defined as the presence of a newly onset focal symptom lasting more than 24 hours which was diagnosed as such by a physician (preferably with the culprit lesion identified/confirmed on CT or MRI/MRA).

C) Onset or progression of nephropathy

Based on urinary albumin (excretion per 1 g of urinary creatinine) classified into

Normoalbuminurea, urinary albumin < 30 mg/g•Cr;
Microalbuminurea, urinary albumin ≥ 30 mg/g•Cr, < 300 mg/g•Cr; and
Macroalbuminurea, urinary albumin ≥ 300 mg/g•Cr,

When either of the following occurs, that time point is to be construed as the occurrence of an event.

a. Progression from normoalbuminurea to microalbuminurea or from normoalbuminurea to macroalbuminurea
b. Progression from microalbuminurea to macroalbuminurea
c. Serum creatinine elevated 2-fold or more compared to that at study entry
d. End-stage renal failure (permanent dialysis initiated or renal transplant performed)

For a. and b., if both the measured urinary albumin value and the value obtained from a retest (using a morning urine sample) are more than 30% higher than that at study entry, the occurrence is considered an event. For c, retesting will be performed within 3 months.

In a., b., or c., the event is to be evaluated in their first occurrence.

Additionally, affected subjects are to be evaluated for annual decreases in glomerular filtration rate as calculated from urinary albumin excretion rate and its conversion formulas.

D) Onset or progression of retinopathy

When either of the following occurs unilaterally or bilaterally, that time point is to be construed as the occurrence of an event.

a. Progression from absence of retinopathy to non-proliferative retinopathy or proliferative retinopathy
b. Progression from non-proliferative to proliferative retinopathy
c. Loss of vision likely due to retinopathy

10.4 Assessment of the endpoints

A separate Endpoint Assessment Committee is to be put in place for macrovascular complications, nephropathy, and retinopathy to verify each of the events that constitute the primary and secondary endpoints of the study.

10.5 Reporting on events

In the event that a new event related to any of the study endpoints was found to have occurred in a particular subject after his/her final study registration, the physician in charge is to report on that event to the Data Center. The Endpoint Assessment Committees are to be convened every 6 months to review each of the events reported in depth.

Even when an event defined as a primary or secondary endpoint occurred in a particular subject, the subject is to continue to be part of the study and observed for occurrence of other events.

11. Safety evaluation

11.1 Technical terminology

In the present study, "adverse reactions" and "side effects" are defined as followed.

- Adverse reactions

  Adverse reactions are defined as any untoward (or unintended) symptoms and signs (including abnormal laboratory findings) that may or may not be causally related to the investigational treatment. In other words, all untoward symptoms or signs that occurred in the study subjects, including those whose causal relationship to the investigational treatment can be ruled out, are to be recorded as "adverse reactions".

  In this trial, all untoward laboratory findings that occurred after the start of the study are to be handled as adverse reactions, in addition to all symptoms and signs that newly occurred or worsened after the start of the study.

- Side effects

  Of the untoward (or unintended) symptoms and signs (including abnormal laboratory findings), those whose causal relationship to the investigational treatment cannot be ruled out are defined as side effects. In other words, of the "adverse reactions" defined above, those whose causal relationship to the investigational treatment cannot be entirely ruled out are to be handled as side effects.

11.2 Collection of data on adverse reactions

During the course of the study, physicians in charge are to collect data on adverse reactions by the following procedures.

Procedure 1:

In interviews with the subjects, encourage them to report spontaneously on any adverse reactions by asking questions (e.g., "Have you had any symptoms that bothered you in the last xx weeks?").

Procedure 2:

Verify if they have had any symptoms through interviews and using checklists. Limit the symptoms to be verified here to the following four symptoms: hypoglycemia, edema, palpitation, and shortness of breath.

Procedure 3:

Identify symptoms not perceived by the subjects by auscultation and percussion. Additionally, identify changes in the laboratory and vital signs data that may be clinically significant or meaningful. The physician in charge may use his/her judgment in deciding which change (e.g., in laboratory findings) may be construed as "clinically meaningful".

The physician in charge is to handle symptoms and signs that newly occurred or worsened after the start of the study as adverse reactions, of all symptoms, signs and changes in laboratory findings, and to deal with them appropriately, to follow up on these symptoms and signs until they disappear, resolve or return to their baseline status, and to describe the name(s) of the adverse reaction(s), date of their occurrence, their extent, their causal relationship to the investigational treatment, their severity, and how they were dealt with (e.g., the investigational drug was reduced or discontinued or switched to another drug), their outcome, and date their outcome confirmed in a case report form.

In the event that the physician in charge judged follow-up unnecessary before these symptoms and signs disappear, resolve or return to their baseline status, he/she is to give reasons for such decision in the case report form.

Rationale for the data collection procedures

These procedures were introduced, on the grounds that the incidence of adverse reactions could vary greatly depending on whether it draws on spontaneous reporting by the subjects (passive surveillance) or on interview- or checklist-based reporting by the physicians (active surveillance) for data collection. In the current multi-center trial, safety surveillance data would not be integrated were it not for the procedures for data collection for use by all participating institutions. While data on adverse reactions are usually collected by passive surveillance in studies such as this one, it was decided that active surveillance be employed in this study to collect data on four major symptoms, namely hypoglycemia (particularly in elderly patients), edema, palpitation and shortness of breath to detect signal symptoms of cardiac failure as serious drug-induced adverse reactions, as the study aims to implement rigorous glycemic control and involves the use of thiazolidinedione derivatives.

References for adverse reaction data collection

1. Food and Drug Administration Center for Drug Evaluation and Research. Reviewer guidance: Conducting a clinical safety review of a new product application and preparing a report on the review (http://www.fda.gov/cider/guidance).
2. Ioannidis JPA, Evans SJW, Gotzsche PC, O'Neil RT, Altman DG, Schulz K, et al. Better reporting of harms in randomized trials: An extension of the CONSORT statement. Ann Intern Med 2004;141:781-788.
3. Friedman LM, Furberg CD, DeMets DL. Fundamentals of clinical trials. 3rd ed. New York: Springer; 1988.

11.3 Severity of adverse reactions and their causal relationship to the investigational treatment

If an adverse reaction occurs, the physician in charge is to rate its severity by using the following three-grade criteria.

Mild	Symptoms or signs are present but the subject experiencing them does not require treatment to continue with the study
Moderate	Symptoms or signs are present but the subject experiencing them calls also for dose reduction of the investigational drug or addition of another drug to continue with the study
Severe	The subject experiencing the symptoms and signs is having difficulty performing daily activities, or his/her clinical course is seriously affected by the symptoms and signs, and the investigational drug needs to be discontinued.

Then, the physician in charge is to rate the casual relationship of the adverse reaction to the investigational drug by using the following four-grade criteria. When the physician in charge judged it to be "unrelated", he/she is to describe the reasons for such judgment.

Unrelated	The adverse reaction can be clearly accounted for by other factors than the investigational drug.
Possibly related	While the adverse reaction is assumed to have resulted from other factors than the investigational drug, its causal relationship cannot entirely be ruled out.
Probably related	The adverse reaction is least likely to have resulted from other factors than the investigational drug.
Definitely related	The adverse reaction is highly likely to have resulted from the investigational drug, e.g., there is a time-dependent association between the time the investigational drug was given and the occurrence of the adverse reaction, or the adverse reaction cannot be accounted for by other reasons than the use of the investigational drug.

All adverse reactions except those defined as "unrelated" are to be handled as side effects.

11.4 Serious adverse reactions

11.4.1 Technical terminology

Any adverse reaction is defined as serious adverse reactions, if it:

Is fatal
Is life-threatening
Leads to the subject becoming hospitalized or calling for prolonged hospitalization or treatment
Leads to permanent or serious damage or dysfunction in the subject
Causes congenital anomaly
Is serious hypoglycemia accompanied by impaired consciousness
Is some other serious medical phenomenon

Or is serious enough to place the subject in a critical situation calling for treatment to avoid the outcomes described above, while it is not immediately fatal, life-threatening or leads to the subject being hospitalized.

11.4.2 Reporting procedure

When a serious adverse reaction occurred, irrespective of its causal relationship to the investigational drug, the physician in charge is to report the adverse reaction to the Trial Secretariat Office within 72 hours of his/her knowledge of its occurrence.

In this regard, the deadline for reporting to the supervisor at his/her own healthcare institution is to be consistent with the regulations laid down by the institution for such reporting. Similarly, all participating healthcare institutions are to report spontaneously to the Pharmaceutical and Food Safety Bureau, Ministry of Health, Labor and Welfare (MHLW), in accordance with the MHLW undertaking, the "Pharmaceuticals and Medical Device Safety Information Reporting System", as well as to relevant pharmaceutical companies in accordance with the "Pharma Reporting System" based on the Pharmaceutical Affairs Law.

11.4.3 Assessment by the Clinical Research Leader and the Trial Secretariat Office

In the event that the Clinical Research Leader receives case reports on adverse reactions, he is to assess their urgency, importance or implications, and to give instructions the Data Center and the participating healthcare institutions of interest, as to their handling, including putting these cases on hold, as required. The Clinical Research Leader may communicate these instructions by telephone depending on the urgency of the cases, followed by those in writing (i.e., by facsimile, mail or e-mail).

The Clinical Research Leader is to communicate his interpretation of the adverse reactions reported as well as their handling (including judgment as to continuation or termination of the study) to the Principal Investigator within 72 hours of their knowledge of occurrence of the adverse reactions, to ask for judgment regarding the validity of their judgment and handling of the cases and forward the "Case Report on Adverse Reactions" sent to them by the reporting healthcare

institutions. Assessment of serious adverse reactions by the Clinical Research Leader should include an evaluation of the each subject's clinical course, as well as of whether or not the frequency of each reported adverse reaction is within their anticipated range. If the Clinical Research Leader judged their frequency to be beyond their anticipated range, the Clinical Research Leader is to document such judgment for reporting.

The Principal Investigator is to submit all reported adverse reactions to the Safety Assessment Committee for review. The Safety Assessment Committee is to review all reported adverse reactions, and to make recommendations to the Principal Investigator with regard to the handling of the cases (including judgment as to whether the study is to be continued with the subjects, instructions are required to the healthcare institutions of interest, or the trial protocol calls for revision). The Principal Investigator is then to give instructions to the Clinical Research Leader in accordance with these recommendations.

11.5 Analysis of adverse reactions through regular monitoring

The Clinical Research Leader and the Trial Secretariat Office are to analyze monitoring reports produced by the Data Center through regular monitoring, and to develop reports on the results of their analysis, where all adverse reactions not reported as urgent are to be evaluated with regard to their category, severity, and rate of occurrence, to determine if they require reporting to the healthcare institutions of interest or call for revision of the trial protocol. At the same time, the Clinical Research Leader and the Data Center are to ensure: that no adverse reactions are left out/missed in the reports submitted by the healthcare institutions; that all reported adverse reactions are entered in regular monitoring reports; and that the results of their analysis include their judgment as to whether there were any adverse reactions left out/missed in the reports received from the participating healthcare institutions.

11.6 Adverse reactions to be anticipated

All investigators are to refer to the package inserts and the product monographs of the drugs to be used in the trial for information on anticipated adverse reactions with the drugs as well as their rate of occurrence.
Specifically, anticipated adverse reactions include hypoglycemia, edema, cardiac failure, gastrointestinal symptoms including nausea/vomiting and discomfort, anemia, palpitation, skin rash, eczema, high CPK values, rhabdomyolysis, lactic acidosis, leucopenia, thrombocytopenia, hyperkalemia, and abnormal GOT/GPT values.

12. Statistical analysis

12.1 Objective

The objective of the statistical analysis is to verify the superiority of intensive therapy over conventional therapy as reference/control. With the primary endpoint defined as the "occurrence of either of myocardial infarction, coronary bypass surgery, percutaneous transluminal coronary angioplasty, stroke, carotid endarterectomy, percutaneous transluminal cerebral angioplasty, carotid artery stenting, or death", the study is designed to verify that intensive therapy is associated with significantly longer event-free survival (time to occurrence of either of myocardial infarction, coronary bypass surgery, percutaneous transluminal coronary angioplasty, stroke, carotid endarterectomy, percutaneous transluminal cerebral angioplasty, carotid artery stenting, or death). In other words, the null hypothesis (H0) of the current trial and its alternative hypothesis (H1) are defined as follows, where when we reject H0 in favor of H1, we conclude that intensive therapy is a useful therapeutic modality.

H0: When the occurrence of either of myocardial infarction, coronary bypass surgery, percutaneous transluminal coronary angioplasty, stroke, carotid endarterectomy, percutaneous transluminal cerebral angioplasty, carotid artery stenting, or death is defined as an event, intensive therapy and conventional therapy are comparable in event-free survival.
H1: When the occurrence of either of myocardial infarction, coronary bypass surgery, percutaneous transluminal coronary angioplasty, stroke, carotid endarterectomy, percutaneous transluminal cerebral angioplasty, carotid artery stenting, or death is defined as an event, intensive therapy is associated with longer event-free survival than conventional therapy.

The secondary endpoints of the study are defined as the following four events.

1. Occurrence of either of myocardial infarction, stroke, or death
2. Onset or progression of nephropathy
3. Occurrence of lower limb vascular events (amputation or revascularization of lower limb)
4. Onset or progression of retinopathy

Of these, onset or progression of nephropathy is also considered an important event; therefore, the current trial is to verify the superiority of intensive therapy over conventional therapy with regard to this event-free survival. In the event that intensive therapy has been proven to be superior to conventional therapy in secondary endpoint 1, we conclude that intensive therapy is a useful therapeutic modality. As the primary and secondary endpoints are not independent of one another, the statistical inference is to be adjusted for multiplicity by using the re-sampling method.
Of note, secondary endpoint 2 was included to complement the primary endpoints. Therefore, only exploratory analysis is to be performed on this endpoint and not to be used as a rationale for the main conclusions to be drawn. In other words, whether or not intensive therapy is superior to conventional therapy in reducing macrovascular complications is to be determined on the basis of an analysis of the primary endpoints.

12.2 Subjects of analysis

The hypotheses for the event-free survivals described above are to be tested in "all subjects judged to be eligible for

inclusion in the study". The decision as to whether or not any particular subject is eligible is to be left up to the designated Clinical Research Leader, the Trial Secretariat Office and the trial statisticians. Additionally, the above hypotheses are also to be tested in "all subjects registered with the study" to confirm the certainty of the conclusions to be drawn.

12.3 Statistical methods

12.3.1 Primary analyses

Descriptive statistics:
When the "occurrence of either of myocardial infarction, coronary bypass surgery, percutaneous transluminal coronary angioplasty, stroke, carotid endarterectomy, percutaneous transluminal cerebral angioplasty, carotid artery stenting, or death" is defined as an event, the event-free survival period (time to occurrence of either of myocardial infarction, coronary bypass surgery, percutaneous transluminal coronary angioplasty, stroke, carotid endarterectomy, percutaneous transluminal cerebral angioplasty, carotid artery stenting, or death) as well as the annual percentage of subjects remaining event-free are to be calculated by the Kaplan-Meier method, and 95% confidence intervals (CI) calculated using Greenwood's formula.

Hypothesis testing:
A stratified log-rank test adjusting the stratification factors used for dynamic allocation (except for participating institutions) is to be used to verify the hypothesis that event-free survival is longer with intensive therapy than conventional therapy, with the "occurrence of either of myocardial infarction, coronary bypass surgery, percutaneous transluminal coronary angioplasty, stroke, carotid endarterectomy, percutaneous transluminal cerebral angioplasty, carotid artery stenting, or death" defined as an event.

Effect size estimates:
Hazard ratios (intensive therapy/conventional therapy) for the events described above as well as 95% confidence intervals are to be calculated by using the Cox proportional hazards model. The adjustment/stratification factors used for dynamic allocation except for participating institutions are to be incorporated into the regression analysis models. Furthermore, if any background factors are found to potentially affect the estimates, these factors are also to be incorporated into the regression analysis models. Details of the approach to be taken are to be described in the Statistical Analysis Plan.

12.3.2 Secondary analyses

Descriptive statistics:
With either one of the secondary endpoints defined as an event, event-free survival (time to occurrence of a secondary endpoint or death), as well as annual percentage of subjects remaining event-free, is to be calculated by using the Kaplan-Meier method, and 95% confidence intervals (CI) are to be calculated by using the Greenwood's formula.

Hypothesis testing:
A stratified log-rank test adjusting the stratification factors used for dynamic allocation (except for participating institutions) is to be used to verify the hypothesis that event-free survival is longer with intensive therapy than conventional therapy, with either one of the secondary endpoints defined as an event.

When intensive therapy has been proven to be superior to conventional therapy in the secondary analyses, we conclude that intensive therapy is a useful treatment modality. However, as the primary and secondary endpoints are not independent of one another, the statistical inference is to be adjusted for multiplicity by using the re-sampling method. Strictly speaking, this adjustment method has the potential to increase Type 1 error for the entire study. However, as it maintains the Type 1 error for the primary endpoints at an appropriate level (5%, two-sided), the adjustment method was deemed valid.

Effect size estimates:
Hazard ratios (intensive therapy/conventional therapy) for the events described above as well as 95% confidence intervals are to be calculated by using the Cox proportional hazards model. The adjustment/stratification factors used for dynamic allocation except for participating institutions are to be incorporated into the regression analysis models. Furthermore, if any background factors are found to potentially affect the estimates, after review of the relevant data with the allocation code blinded, these factors are also to be incorporated into the regression analysis models.

12.3.3 Other analyses

To complement analyses of the primary endpoints, three kinds of analyses (descriptive statistics, hypothesis testing and effect size estimates) are to be performed on the secondary endpoints. However, being exploratory in nature, these analyses are not to be adjusted for multiplicity.

Fisher's direct exact probability estimation is to be used to compare the percentages of occurrence of adverse reactions between the two treatment arms as part of the safety evaluation, and 95% confidence intervals (CI) for the differences detected. Accurate confidence intervals are to be estimated based on the bimodal distribution of the data.

Otherwise, exploratory analyses are to be performed as required. When such analyses are to be performed, their details

are to be described in the Statistical Analysis Plan.

12.4 Interim Analyses and the Clinical Trial Review Committee

12.4.1 Interim analyses

To assess whether the main objectives of the study have been achieved, 3 interim analyses were planned as follows. These analyses were to be performed by independent statistical analysts at the Data Center.

- Early after completion of the subject study registration
- 1 year after completion of the subject study registration
- 2 years after completion of the subject study registration

However, as the general survey conducted in June 2006 demonstrates that fewer subjects had been accrued to date and fewer events were reported than initially expected, interim analyses are to be performed based the nine general surveys scheduled to place at the following time points:

- December 2007
- December 2008
- December 2009
- December 2010
- June 2011
- June 2012
- June 2013
- June 2014
- June 2015

Data reviewed by the Endpoint Assessment Committee are to be used for analysis at each of the interim analyses. Unless otherwise notified, the study subjects are to continue their study registration even during the interim analyses. Once the interim analysis results have demonstrated that the main objectives of the study have been achieved, the trial is to be discontinued and the study results to be released.

To reduce the Type 1 error associated with the multiple analyses (interim and final analyses) and to maintain the two-sided Type 1 error at 5%, analyses of the primary endpoints are to be adjusted for multiplicity by using a Lan-DeMets alpha function approach with an alpha -spending function of the O'Brien-Fleming type. In addition, Beyesian Probability or conditional power is to be calculated as reference data.

Of the secondary endpoints, "time to onset or progression of nephropathy or occurrence of death" is to be analyzed with adjustment by the re-sampling method for multiplicity in relation to the primary endpoints. No inter-group comparisons are to be made except this endpoint, unless the Trial Assessment Committee or the Central Ethics Committee approves such comparison.

In the event that the interim analysis results have demonstrated the superiority of intensive therapy over conventional therapy, as a general rule, the trial is to be discontinued. When the event rate has been shown to be higher with intensive therapy than with conventional therapy, the hypothesis is not to be tested, and the overall decision as to the termination of the trial is to be made.

A detailed Interim Statistical Analysis Plan is to be jointly developed by the Clinical Research Leader, Trial Secretariat Office, trial statisticians and independent statistical analysts through mutual consultation. The independent statistical analysts are not to be involved in the revision of the Statistical Analysis Plan or the Patient Accrual Goal that may occur after the first interim analysis.

12.4.2 Reporting on/review of statistical analyses

The independent statistical analysts are to draft and submit the interim analysis reports to the Trial Assessment Committee, which is in turn to make its decision as to the continuation of the study, and to make its recommendations to the Principal Investigator, the Foundation Secretariat, and the Clinical Research Leader.

Unless the Trial Assessment recommends discontinuing the study, no personnel except for the Trial Assessment Committee is to be given access to the results of the interim analyses. The Trial Assessment Committee is obliged not to disclose the interim analysis data to any personnel outside the Trial Assessment Committee. However, the Clinical Research Leader is to be construed as forming part of the Trial Assessment Committee.

The Clinical Research Leader is to make the decision as to whether the trial is to be continued or discontinued or the trial protocol is to be revised in part, on the recommendation of the Trial Assessment Committee. In the event that the trial is to be discontinued or the trial protocol is to be revised in part, the Clinical Research Leader is to submit a "request for early terminnation of the trial" or "request for the revision of the trial protocol" in writing. The Operations Committee is to review the request, and to obtain the approval of the Central Ethics Committee if the Trial Administrative Committee is to decide to revise the trial protocol in part. Upon approval by the Central Ethics Committee, the Clinical Research Leader is to be entitled to revise the trial protocol as requested.

The Clinical Research Leader is to be entitled to raise an objection to the recommendations of the Trial Assessment Committee. The Trial Assessment Committee is to be entitled to make its recommendations again after deliberations. In the event of failure to harmonize the views between the Clinical Research Leader and the Trial Assessment Committee, The Trial Administrative Committee is to make a final decision as to the continuance or discontinuance of the trial or revision of the trial protocol.

12.4.3 Follow-up in the event of early trial termination

In the event that the trial is to be terminated in light of interim analysis results, the study subjects are to be followed up until March 2016.

12.4.4 Final analyses

After the follow-up period, the study data are to be consolidated for final analysis. Results of the final analysis are to be compiled by the Data Center as a final analysis report for submission to the Clinical Research Leader, the Trial Secretariat Office, the Trial Administrative Committee, and the Trial Assessment Committee.

The Clinical Research Leader and the Trial Secretariat are to review the final analysis report and to draft a "Final Clinical Research Report" that describes the conclusions of the entire study, issues and problems, interpretation and discussion of the study results, and future prospects and issues from a primarily clinical point of view for submission to the Trial Administrative Committee, Central Ethics Committee, and Trial Assessment Committee. The current trial is to be construed as terminated when the Final Research Report has been approved by the Trial Administrative Committee.

13. Target patient accrual and the rationale for the target setting

The target patient accrual is projected at 1,669 each for intensive and conventional therapy, totaling 3,338 patients involving a total of 81 healthcare institutions that each allows 50 or more subjects to be accrued.

The target patient accrual number was calculated on the following rationale.

The trial subjects were estimated to include those with ischemic heart disease and those without at a ratio of 7:3, with the occurrence of events including deaths per year estimated as 4.4% (10.0% in those with a history of ischemic heart disease and 2.0% in those without) in the conventional therapy arm versus 3.08% (7.063% in those with a history of ischemic heart disease and 1.395% in those without) in the intensive therapy arm.

Based on this estimate, under the assumption that the subjects are to be accrued over a period of 1 year for follow-up for 2 years and 9 months, the number of patients required for this trial was calculated by using the Shoenfeld-Richter nomograms as 1,408 subjects per arm with the expected number of events being 328, to verify that intensive therapy is superior to conventional therapy at the two-sided significance level of 5% with a 90% power. In light of the results of the general survey conducted in June 2007, the annual event rate was revised to about 1/2 of the original estimate, with the hazard ratio estimated, as previously estimated, as 2.2% in the conventional therapy arm versus 1.5347% in the intensive therapy arm. With the patient accrual extended to December 2008 (with an accrual period of 2.5 years) and follow-up extended to March 2013 (with a follow-up period of 4.25 years) based on this estimate, the expected number of events remained the same, and the number of subjects required for both arms were estimated to total 2,338.

However, in the event that the expected number of events is to be judged unattainable given the pace at which events occurred in both arms at the time of regular monitoring by the Data Center, the Trial Secretariat is to consult the trial statisticians and file a request for revision of the trial protocol with the Central Ethics Committee, where revision of the trial protocol is to be construed as either of the following:

① Extension of the patient accrual period or the follow-up period
② Revision upward of the patient accrual number

The final number of subjects who enrolled in the study during the registration period was 2,542 patients. Based on a patient accrual period of 2.5 years with a follow-up period of 4.25 years, the power of the test will be ≥ 80% with a 5% level of significance (2-sided). Therefore, the expected number of events will be changed to 250 because an interim analysis aimed at demonstrating the superiority of intensive therapy over conventional therapy in order to discontinue the trial, has not been conducted.

The rate of occurrence was lower than what was expected before the start of the study, suggesting the number of events would not reach the required number of events during the predefined follow-up period. Since the registration period was expired, addition of subjects was impossible. Thus, follow-up period was extended for a sufficient time until the required number of events could be obtained.

14. Ethical considerations

14.1 Observance of the ethical criteria

The current trial is to be conducted in accordance with the Declarations of Helsinki and the "Ethical Guidelines for Medical and Health Research Involving Human Subjects." All of the investigators and sub-investigators responsible for the trial must comply with the ethical standards stated above.

14.2 Ethics Review Committees

Prior to the conduct of the study, the Ethics Review Committees of all participating healthcare institutions (committees jointly set up in collaboration with the heads of other participating institutions if any participating institution is incapable of setting one up on its own or committees set up by public interest corporations in accordance with article 34, Civil Law) are to review the current study for its feasibility as well as for its ethical and scientific validity. The current trial is to be conducted after approval of these Ethics Review Committees.

If any Ethics Review Committee recommended approval pending some revision l, only where these revisions have nothing to do with the inclusion criteria, the intended therapeutic interventions or the study endpoints, these revisions are to be considered in mutual consultation with the Clinical Research Leader for inclusion, those revisions considered

appropriate based on the results of deliberations are to be incorporated in the trial-related documents such as trial protocol or the patient informed consent form, and the trial is to be conducted accordingly.

In addition, the Ethics Review Committees are to continually review the trial status to see if it is being conducted in accordance with the ethical criteria once a year or more frequently.

14.3 Patient informed consent

Each trial investigator is to explain to all candidate subjects regarding the following prior to their participation in the study:

1) That their participation is put on a voluntary basis;
2) That their not giving consent to participate does not lead to any disadvantage on their part;
3) That they are able to withdraw their consent (for subjects who are incompetent, consent from the family member or legally authorized representative) at any time and this does not lead to any disadvantage on their part;
Withdrawal of consent means here ① that while they discontinue the investigational treatment, they allow data to be generated on subsequent follow-up to be provided for analysis and ② that they withdraw their consent and refuse to provide the follow-up data, and all subjects are able to choose between these two options;
4) The reason that they were asked to participate in the study, their disease name and anticipated prognosis;
5) The details of the trial they participate in (the rationale for its conduct, its objectives, details of the planned investigational treatment, and the study duration);
6) The name and title of the investigator
7) That this study represents a "clinical trial" based on the Ministry of Health, Labor and Welfare's strategic outcome research and how it differs from usual medical consultation;
8) The benefits potentially associated with participation in the study (note: this does not mean the estimated "effect" of the investigational treatment);
9) The potential disadvantages or inconveniences potentially associated with participation in the study, as well as anticipated adverse reactions and how they are to be dealt with when they appear;
10) Cost burden and compensation associated with participation in the study
All treatment-associated costs are to be covered by the insurance system, and the compensation for any health disturbance is to be made in a similar fashion to that in usual medical consultation;
11) If the subject so desires, he/she is to be given access to the trial information such as the trial protocol, so long as it does not affect the maintenance of the other subjects' privacy or the originality of the intended trial;
12) The treatment to be offered if he/she decides not to participate in the study;
13) Handling of personal information, and where it is to be provided
That utmost care is to be exercised in keeping the personal information confidential, and that the personal information (medical record number, his/her date of birth as well as initials of his/her name) is to be used to ensure accurate identification of the subject due to the need to collect his/her clinical data on a continuous basis during the course of the study, and that the personal information is to disclosed to the Data Center only but his/her identity such as his/her name cannot be established on the basis of this data.
That if the subject changed hospital, the Data Center may collect relevant clinical data with the consent (for subjects who are incompetent, proxy consent from the family member or legally authorized representative) of the subject and that if the Data Center is unable to contact him/her, the Data Center is to consider sending a Clinical Research Leader-designated agent to find out whether or not the subject is dead or alive where he/she lives by consulting the Basic Residence Register. Additionally, if permission is obtained to do so from the Ministry of Internal Affairs and Communications, the Data Center is to consider sending a Clinical Research Leader-designated agent to look into the cause of death of the subject by consulting the Vital Statistics.
That his/her medical record is to be reviewed at the time of monitoring by the Data Center or for auditing purposes, in which case, however, the personal information is to be kept strictly confidential;
14) The outcome of the study is to be released in such a way that the study subjects cannot be identified;
15) Secondary use of the clinical data
That with regard to the subject's clinical data, only when it is reviewed and approved by the Trial Administrative Committee, it may be used for other studies (note: e.g., meta-analyses), and in which case all information that leads to his/her identity being established will be removed from the data to be provided;
16) The point of contact for queries or complaints by the participating subject

After the investigator has explained the above to the subject and confirmed that the subject had an understanding of the details of the study he/she is to participate in, the investigator is to inquire the subject one or more days after all this explanation as to his/her consent to participate in the study. If the subject is to consent to participate in the study, the investigator is to obtain his/her consent in writing by using an informed consent form to be prepared as a separate document (or a version of this informed consent that has been revised by the relevant participating healthcare institution and that has been approved by the relevant Ethics Review Committee). The informed consent form is to include the name of the investigator who has explained and obtained the consent, the date of the consent obtained, and to be signed up by the subject and the investigator.

The informed consent form is to be photocopied in duplicate, so that one copy is to be retained by the subject, and the other to be retained by the relevant healthcare institution. The original copy of the informed consent is to be kept on file with the medical record of the subject.

14.4 Protection of privacy and identification of the study subjects

The Data Center is to be informed of the subject's personal information by the relevant healthcare institution at the time of his/her study entry. However, inquiries from the Data Center are to be made by using the entry number for the subject issued at his/her study entry and the medical record number so that it does not allow any third party who may gain access these communications to establish the identity of the subject directly.

While the use of the medical record number always involves the potential risk of leakage of the personal information, the non-use of the medical record number leads to sole dependence on the personal data management by the relevant healthcare institution for establishing the identity of a particular subject. In the current trial with long follow-up in which the subjects may go through changes of address or hospital, sole reliance on the healthcare institution for personal data management may likely lead to the identification of their identity becoming impossible. With the increasing number of the subjects lost to follow-up, the authenticity of the study results may be so jeopardized that they may not be reflected in the relevant healthcare policy by the Ministry of Health, Labor and Welfare.

Given the above considerations, it is decided that the Data Center is to handle all personal information on the participating subjects that allows these subjects to be established. Additionally, all trial investigators are to pay utmost care in the handling of the subjects' personal information, and to explain to the subjects beforehand how much of their personal information is to be handled and how it is to be utilized. All communications between the participating healthcare institutions, the Data Center and the Trial Secretariat are to be conducted by post or to be hand-delivered as a general rule, irrespective of whether they contain hard copy or electronic material. E-mail sending of personal information without the use of an appropriate security measure such as encrypted codes is to be prohibited.

15. Quality management

15.1 Procedures for case report development and patient data consolidation

A series of procedures leading from the development of case reports by the physician in charge through keying in of the data by the Data Center to the consolidation of the clinical data are to be described separately in the standard operating procedure (SOP).

15.2 Data monitoring

Details of the data monitoring to be conducted by the Data Center as well as the procedures involved in data monitoring are to be described separately in the standard operating procedure (SOP).

15.3 Auditing

In the event that a separate audit is to be implemented apart from the data monitoring described above, details of the planned audit, the material it is intended to review as well as the procedures involved in such auditing are to be described separately in the standard operating procedure (SOP).

16. Data handling and archiving

All participating healthcare institutions are obliged to appropriately retain all of the documents that the Clinical Research Leader and relevant Ethics Review Committees decide to archive until the day on which 5 years have elapsed from the date of report of study completion or the day on which 3 years have elapsed from report of final publication of study results, whichever comes later.

17. Policy for data disclosure

All participating physicians or nurses are to consult the Principal Investigator and the Clinical Research Leader beforehand when releasing the outcomes results of the study or findings from the study.

18. Violations and revisions of the trial protocol

18.1 Violation of the protocol to avoid emergencies and crises

For reasons that are medically compelling such as avoidance of an emergency or crisis in a particular subject, the physician in charge may violate or adapt the trial protocol without prior approval by the intramural Ethics Review Committee.

In the case of such violation or adaptation of the trial protocol for reasons that were medically compelling, the physician in charge is to report to the Trial Secretariat, the Data Center and the Ethics Review Committee as soon as possible about details of the violation or adaptation of the trial protocol thus implemented as well as the reasons for such violation and adaptation, asking for approval of such decision. At the same time, the physician in charge is to record the details of the violation or adaptation in the case report form.

18.2 Revision of the trial protocol and early termination of the trial

The procedures for early termination of the trial or revision of the trial protocol following the emergence of a serious safety concern are described in section 11.4, and those for revision of the trial protocol or early termination of the trial in light of interim analysis results are described in section 12.4.

When the Clinical Research Leader judged the trial protocol to require revision or the study to require early termination for other reasons, they are to decide whether not to revise the protocol or terminate the trial in mutual consultation with the Trial Administrative Committee, the Trial Assessment Committee, the Safety Evaluation Committee, and the Central

Ethics Committee, and to communicate their decision to the healthcare institutions and physicians in charge as soon as possible.
When revising the trial protocol for either of the above reasons, the physician in charge at each participating healthcare institution is to submit the revised protocol to the relevant Ethics Review Committee for review and approval. At the same time, when the physician in charge judges the revision of the patient informed consent form in conjunction with the revision of the protocol, he/she is to revise the patient informed consent and obtain informed consent from the subjects who gave their informed consent (for subjects who are incompetent, proxy consent from the family member or legally authorized representative) before revision of the informed consent form.

19. Trial investigators and committees

19.1 Principal Investigator
The person designated by the Japan Diabetes Foundation (before March 2013, the Director of Japan Foundation for the Promotion of international Medical Research Cooperation [JF-PMIRC])

19.2 Clinical Research Leader
Takashi Kadowaki, Department of Diabetes and Metabolism, Graduate School of Medicine, University of Tokyo

19.3 Trial Coordinating Committee
The Clinical Research Leader may delegate to the Trial Coordinating Committee authority in coordinating the interpretation of the trial protocol and other minor details of the study.
The coordination the Clinical Research Leader may delegate to the Trial Coordinating Committee includes the following:
1) Coordination among the participating healthcare institutions about the minor details of the trial protocol
2) Harmonization of potential conflicting interpretation of the trial protocol during conduct of the trial
3) Coordination among the participating healthcare institutions

The Trial Coordinating Committee is to coordinate among the healthcare institutions about the minor details of the trial protocol based on opinion and feedback gathered from these institutions. Additionally, when the trial protocol calls for revision, the Trial Coordinating Committee is to receive feedback on the proposed revision of the trial protocol from the participating institutions and to make the revised protocol definitive in light of this feedback.

19.4 Trial Administrative Committee
The Trial Administrative Committee is to be composed of 15 committee members to be appointed by Director, JF-PMIRC, who provide consultation and make recommendations regarding the following in response to requests by Director, JF-PMIRC:
(1) Evaluation of researcher participant applicants for the strategic outcome research
(2) Alignment of the trial conduct infrastructure
(3) Planning of the strategic outcome research plan
(4) Budgeting and audit of the strategic outcome research project
(5) Other fundamental issues in the implementation of the strategic research project

The Chairperson of the Trial Administrative Committee is to be appointed by Director, JF-PMIRC.

The Director of the JF-PMIRC is to report to the Trial Administrative Committee on the results of deliberations and notifications by all the trial committees (except for the Trial Administrative Committee).

19.5 Trial Assessment Committee
The Trial Assessment Committee is to provide interim reports (evaluations of interim analyses) on the progression of the trial in view of the recommendations it is to make regarding the continuation of the trial or release of the study results while being in close contact with the Trial Progress Management, and to make recommendations regarding the measures and actions to be taken to the Principal Investigator, the JD-PMIRC Secretariat, and the Clinical Research Leader.
The Chairperson of the Committee is to be chosen by the members of the Committee from among the members of the Committee. The Chairperson of the Committee is to convene members of the Committee and to serve as Chairperson. Meetings by the Committee are to be held with the JF-PMIRC Secretariat, Trial Progress Management members, the Clinical Research Leader and Clinical Research Leader-appointed personnel (including the independent statistical analyst) attending, in addition to the members of the Trial Assessment Committee.
The Chairperson is to appoint a committee member in charge of the meeting minutes prior to the meeting, where the Chairperson may combine this role. Prior to deliberations of a particular research issue, the Clinical Research Leader is to provide an outline of the issue, along with any new developments with the issue. Members of the Trial Progress Management are to report on the status of trial monitoring. Prior to deliberations, all members of the Trial Assessment Committee and the independent statistical analyst are to excuse themselves, and the independent statistical analyst is to account for the interim analysis results and evaluate the results. The Trial Assessment Committee is to make its recommendations as to the "early termination" or "continuation" of the study after its consensual decision making. If the decision was divided, the decision is to be made by a majority vote, where in the case of a tie the Chairperson is to cast a tie-breaking vote. The committee member in charge of the meeting minutes is to summarize the results of deliberations,

and to seal these minutes together with the interim analysis results. After deliberations, the Committee is to notify the Principal Investigator, the JF-PMIRC Secretariat, and the Clinical Research Leader of their recommendations. The interim analysis results and the minutes of the Committee deliberations thus sealed are not to be unsealed for disclosure until trial termination is recommended or until the completion of the final analysis.

19.6 Trial Progress Management

The Trial Progress Management is to audit monitoring of the clinical trial except in the interim analyses. This monitoring refers to central monitoring as conducted via the Data Center and on-site monitoring in which the monitor is to visit a particular participating healthcare institution for inspection.

The Chairperson of the Committee is to be appointed by Director, JF-PMIRC.

The Trial Progress Management may be run by means of consensual decision making through teleconferencing, communications by post, e-mails, in addition to on-site meetings.

The Trial Progress Management is to receive as month updates from the Data Center on the patient accrual status, patient eligibility for study entry, appropriateness of subject allocation, the presence or absence of trial violations, the incidence of serious adverse reactions, and the occurrence of endpoints with the treatment arms masked. The Trial Progress Management is to evaluate whether the study is being performed in a safe and appropriate fashion, and to report to the Principal Investigator on the results of their deliberations.

If there occurred a problem with regard to the progress of the study that might affect the science and ethics of the study, the Trial Progress Management is to report to the Principal Investigator and the Clinical Research Leader to that effect, and to discuss solutions to the problem with the relevant research groups and research support organizations.

19.7 Safety Assessment Committee

In response to requests from the Principal Investigator, the Safety Assessment Committee is to discuss the measures and actions to be taken as well as the casual relationship with the investigational treatment in regard to the adverse reactions for which emergency reporting is designated, and to report to the Principal Investigator.

The Chairperson of the Committee is to be appointed by Director, JF-PMIRC.

When an adverse reaction is reported that require evaluation by the Safety Assessment Committee, the Principal Investigator is to make an immediate request to the Committee Secretariat to review the adverse reaction, and the Committee Secretariat is to communicate the reported status to 2 or more members of the Committee by post or by parcel delivery service.

When the Principal Investigator and the Safety Assessment Committee Secretariat found the reported details inadequate, they may ask the reporting physician for a greater account of the adverse reaction reported.

Upon request from the Principal Investigator, the Safety Assessment Committee is to provide the results of their deliberations on the case submitted, and the Chairperson is to summarize these deliberations. When the Chairperson found the deliberations to possibly affect the decision as to the continuation of the study or call for revision of the trial protocol, he/she is to report to the Principal Investigator to that effect immediately. When the above considerations do not apply, the Chairperson is to report to the Principal Investigator in his monthly reports.

When the Principal Investigator received a report from the Safety Assessment Committee on an adverse reaction reported whose causal relationship with the investigational treatment could not be denied, the Principal Investigator is to communicate the review this judgment to the Clinical Research Leader and the Trial Secretariat immediately, and give instructions on the measures and actions to be taken.

19.8 Central Ethics Committee

Japan Diabetes Foundation (before March 2013, JF-PMIRC) is to set up the Central Ethics Committee to ensure the conduct of the medical research in accordance with the Helsinki declarations.

The Central Ethics Committee is to be comprised of the following personnel to be appointed by Director, JF-PMIRC.

- 1 Chairperson
- Members of the Committee including those that are given as follows:

1) 2 or more experts in clinical trials
2) 2 or more medical experts
3) Non-medical experts
    1 or more experts in humanities and social sciences such as experts in legal science
    1 or more representatives of the general public
4) Other personnel to be appointed by the Director of the JF-PMIRC.

The Central Ethics Committee is to review the ethical and scientific validity of any medical research that comes within its purview at the request of the Director of the JF-PMIRC, and to make relevant recommendations in writing. Additionally, the Central Ethics Committee is to review reports by the Trial Assessment Committee, the Safety Assessment Committee, the Trial Progress Management and the intramural Ethics Review Committee of the participating healthcare institutions as to their ethical and scientific validity, and to make recommendations regarding the continuation, revision, or early termination of the study.

20. Research funding

Research funding is to be accounted for by Ministry of Health, Labor and Welfare grants in aid and corporate donations (described elsewhere). All research funds are to be obtained through the Japan Diabetes Foundation and donated funds are to be used for establishing infrastructure for the study at each healthcare institution. All devices for self-monitoring of

blood glucose (SMBG) will be supplied at no charge by a manufacturer (described in a separate document). Manufacturers who donate funds or supply resources at no charge will never be involved in the conduct, analysis, or report of the study.

21. Conflicts of interest

The Principal Investigator and the Clinical Research Leader are to declare conflicts of interest (described in a separate document) to the healthcare institution yearly. In addition, the investigator at each healthcare institution is to declare conflicts of interest to his/her healthcare institution and the Principal Investigator yearly for matters that should be disclosed (including information about the sub-investigators, but excluding amounts of money). At the beginning of each fiscal year, the Principal Investigator is to confirm based on these declarations whether or not any new "conflicts of interest" that may affect study results or interpretation of these results have occurred with respect to the design, conduct, or reporting of the study, and that the conduct of the study will never impair subject rights and benefits.
