# Supplementary material for: Effect of a Multifactorial Intervention on Retinopathy in People With Type 2 Diabetes: A Secondary Analysis of the J-DOIT3 Randomized Clinical Trial
Source: JAMA Ophthalmol. 2025 Oct 23;143(12):989–97. doi: 10.1001/jamaophthalmol.2025.3819 (PMC12550739; doi:10.1001/jamaophthalmol.2025.3819)
Supplement: Supplement 2. — eTable 1. Baseline Characteristics eTable 2. The Number of the First Retinopathy Events Stratified by Retinopathy at Baseline eTable 3. Multivariable Cox Regression Analysis of Onset or Progression of Retinopathy With Baseline Characteristics as Covariates eTable 4. Multivariable Cox Regression Analysis of Onset of Retinopathy With Baseline Characteristics as Covariates eTable 5. Multivariable Cox Regression Analysis of Progression of Retinopathy With Baseline Characteristics as Covariates eTable 6. HbA1C Change During the First Year of Intervention and Hypoglycemia eTable 7. Yearly Incidence of Hypoglycemia During the Intervention Period eFigure 1. Effects of Hypoglycemia on Onset of Retinopathy in Both Therapy Groups eFigure 2. Effects of Hypoglycemia on Onset of Retinopathy in Participants Without Receiving a Blood Glucose Meter [file jamaophthalmol-e253819-s002.pdf]

Supplemental Online Content

Sasako T, Ueki K, Miyoshi K, et al; J-DOIT3 Study Group. Effect of a multifactorial intervention on retinopathy in people with type 2 diabetes: a secondary analysis of the J-DOIT3 randomized clinical trial. *JAMA Ophthalmol*. Published online October 23, 2025. doi:10.1001/jamaophthalmol.2025.3819

eTable 1. Baseline Characteristics
eTable 2. The Number of the First Retinopathy Events Stratified by Retinopathy at Baseline
eTable 3. Multivariable Cox Regression Analysis of Onset or Progression of Retinopathy With Baseline Characteristics as Covariates
eTable 4. Multivariable Cox Regression Analysis of Onset of Retinopathy With Baseline Characteristics as Covariates
eTable 5. Multivariable Cox Regression Analysis of Progression of Retinopathy With Baseline Characteristics as Covariates
eTable 6. HbA_{1c} Change During the First Year of Intervention and Hypoglycemia
eTable 7. Yearly Incidence of Hypoglycemia During the Intervention Period
eFigure 1. Effects of Hypoglycemia on Onset of Retinopathy in Both Therapy Groups
eFigure 2. Effects of Hypoglycemia on Onset of Retinopathy in Participants Without Receiving a Blood Glucose Meter

This supplemental material has been provided by the authors to give readers additional information about their work.

© 2025 Sasako T et al. *JAMA Ophthalmology*.

eTable 1. Baseline Characteristics

Characteristic	Conventional (N = 1271)	Intensive (N = 1269)
Age [year] †	59.1±6.3	58.9±6.4
Female sex †	480 (37.8)	485 (38.2)
Duration of diabetes [year]	8.47±6.99	8.58±7.00
Smoking status		
Current	267 (21.0)	328 (25.8)
Former	416 (32.7)	370 (29.2)
Never	588 (46.3)	571 (45.0)
History of CVD †	142 (11.2)	146 (11.5)
Body weight [kg]	65.9±12.0	65.4±11.9
Body mass index [kg/m^2]	24.9±3.8	24.8±3.6
Fasting plasma glucose [mg/dL]	158.7±39.4	159.6±41.5
HbA_{1C} [%] †	7.98±1.05	8.01±1.05
HbA_{1C} [mmol/mol] †	63.7±11.5	64.0±11.5
Systolic blood pressure [mm Hg]	134.1±16.3	133.5±16.9
Diastolic blood pressure [mm Hg]	80.0±11.1	79.3±10.8
LDL cholesterol [mg/dL]	125.6±31.7	125.5±30.6
HDL cholesterol [mg/dL]	54.5±14.0	54.4±14.9
Triglyceride [mg/dL]	123 (88, 177)	121 (85, 180)
Urine ACR [mg/g･Cr]	10.8 (5.8, 26.3)	11.1 (5.9, 27.6)
Urine ACR ≥ 30mg/g･Cr	289 (22.7)	297 (23.4)
eGFR [mL/min/1.73m^2]	82.2±18.1	82.3±17.8

© 2025 Sasako T et al. *JAMA Ophthalmology*.

eGFR < 60mL/min/1.73m^2	110 (8.7)	103 (8.1)
Retinopathy		
None	1055 (83.0)	1042 (82.1)
Non-proliferative, one eye	47 (3.7)	45 (3.5)
Non-proliferative, both eyes	169 (13.3)	182 (14.3)

Data are mean ± SD, N (%), or median (IQR). † Dynamic allocation was performed by randomization adjusted for age, male-to-female ratio, history of cardiovascular disease, and HbA_{1C}. CVD, cardiovascular disease; LDL, low-density lipoprotein; HDL, high-density lipoprotein; ACR, albumin-creatinine ratio; eGFR, estimated glomerular filtration rate.

© 2025 Sasako T et al. *JAMA Ophthalmology*.

eTable 2. The Number of the First Retinopathy Events Stratified by Retinopathy at Baseline

Outcome	Retinopathy at baseline	First retinopathy event	Conventional (362 events)	Intensive (317 events)
Onset	None	Non-proliferative or proliferative retinopathy in at least one eye	311	261
Progression	Non-proliferative, one eye	Non-proliferative or proliferative retinopathy in the healthy eye	25	27
		Proliferative retinopathy in the affected eye	2	2
	Non-proliferative, both eyes	Proliferative retinopathy in at least one eye	24	27

First retinopathy events were broken down into onset or progression depending on the presence or absence of non-proliferative retinopathy at baseline. No blindness occurred in either group.

© 2025 Sasako T et al. *JAMA Ophthalmology*.

eTable 3. Multivariable Cox Regression Analysis of Onset or Progression of Retinopathy With Baseline Characteristics as Covariates

Explanatory Variables		Regression Coefficient (95% CI)	P Value
Treatment group	Conventional / Intensive	0.85 (0.73 - 0.99)	.04
Age	＜60 years / ≥ 60 years	1.03 (0.88 - 1.21)	.73
Sex	Male / Female	1.08 (0.88 - 1.32)	.46
History of CVD	No / Yes	0.98 (0.76 - 1.25)	.84
HbA_{1C}	＜8.9% / ≥8.9%	1.16 (0.94 - 1.44)	.16
Body mass index	＜25 / ≥ 25	0.82 (0.69 - 0.96)	.02
Smoking status	Never / Current or former	0.97 (0.80 - 1.18)	.75
Duration of diabetes	＜10 years / ≥ 10 years	1.41 (1.20 - 1.66)	<.001
Fasting plasma glucose	＜180 mg/dL / ≥ 180 mg/dL	1.33 (1.10 - 1.61)	.003
Systolic blood pressure	＜130 mmHg / ≥ 130 mmHg	1.19 (0.99 - 1.43)	.07
Diastolic blood pressure	＜80 mmHg / ≥ 80 mmHg	1.18 (0.99 - 1.41)	.06

© 2025 Sasako T et al. *JAMA Ophthalmology*.

LDL cholesterol	＜120 mg/dL / ≥ 120 mg/dL	1.04 (0.89 - 1.22)	.61
HDL cholesterol	＜40 mg/dL / ≥ 40 mg/dL	0.83 (0.65 - 1.05)	.12
Triglyceride	＜150 mg/dL / ≥ 150 mg/dL	0.98 (0.83 - 1.16)	.79
Urine ACR	＜30mg/g･Cr / ≥ 30mg/g･Cr	1.24 (1.04 - 1.48)	.02
eGFR	＜60mL/min/1.73m^2 / ≥ 60mL/min/1.73m^2	1.17 (0.87 - 1.56)	.31

Multivariable Cox regression for onset or progression of retinopathy with allocation factors and pre-specified factors at baseline. Covariates were available in 2421 participants and 659 events were observed. The referred category is indicated in the left. CVD, cardiovascular disease; LDL, low-density lipoprotein; HDL, high-density lipoprotein; ACR, albumin-creatinine ratio; eGFR, estimated glomerular filtration rate.

© 2025 Sasako T et al. *JAMA Ophthalmology*.

eTable 4. Multivariable Cox Regression Analysis of Onset of Retinopathy With Baseline Characteristics as Covariates

Explanatory Variables		Regression Coefficient (95% CI)	P Value
Treatment group	Conventional / Intensive	0.83 (0.70 - 0.99)	.03
Age	＜60 years / ≥ 60 years	1.02 (0.86 - 1.22)	.81
Sex	Male / Female	1.05 (0.85 - 1.31)	.65
History of CVD	No / Yes	0.98 (0.75 - 1.30)	.90
HbA_{1C}	＜8.9% / ≥8.9%	1.20 (0.95 - 1.51)	.13
Body mass index	＜25 / ≥ 25	0.76 (0.64 - 0.91)	.003
Smoking status	Never / Current or former	0.94 (0.76 - 1.16)	.55
Duration of diabetes	＜10 years / ≥ 10 years	1.50 (1.25 - 1.79)	<.001
Fasting plasma glucose	＜180 mg/dL / ≥ 180 mg/dL	1.45 (1.18 - 1.78)	<.001
Systolic blood pressure	＜130 mmHg / ≥ 130 mmHg	1.29 (1.05 - 1.59)	.01
Diastolic blood pressure	＜80 mmHg / ≥ 80 mmHg	1.13 (0.93 - 1.37)	.23

© 2025 Sasako T et al. *JAMA Ophthalmology*.

LDL cholesterol	＜120 mg/dL / ≥ 120 mg/dL	1.04 (0.88 - 1.24)	.64
HDL cholesterol	＜40 mg/dL / ≥ 40 mg/dL	0.84 (0.64 - 1.10)	.20
Triglyceride	＜150 mg/dL / ≥ 150 mg/dL	0.97 (0.81 - 1.16)	.71
Urine ACR	＜30mg/g･Cr / ≥ 30mg/g･Cr	1.27 (1.04 - 1.56)	.02
eGFR	＜60mL/min/1.73m^2 / ≥ 60mL/min/1.73m^2	1.03 (0.74 - 1.42)	.88

Multivariable Cox regression for onset of retinopathy in participants without retinopathy at baseline with allocation factors and pre-specified factors at baseline. Covariates were available in 1993 participants and 553 events were observed. The referred category is indicated in the left. CVD, cardiovascular disease; LDL, low-density lipoprotein; HDL, high-density lipoprotein; ACR, albumin-creatinine ratio; eGFR, estimated glomerular filtration rate.

© 2025 Sasako T et al. *JAMA Ophthalmology*.

eTable 5. Multivariable Cox Regression Analysis of Progression of Retinopathy With Baseline Characteristics as Covariates

Explanatory Variables		Regression Coefficient (95% CI)	P Value
Treatment group	Conventional / Intensive	1.01 (0.69 - 1.48)	.97
Age	＜60 years / ≥ 60 years	0.95 (0.63 - 1.44)	.82
Sex	Male / Female	1.37 (0.80 - 2.34)	.26
History of CVD	No / Yes	1.04 (0.61 - 1.80)	.88
HbA_{1C}	＜8.9% / ≥8.9%	1.17 (0.70 - 1.95)	.54
Body mass index	＜25 / ≥ 25	1.08 (0.71 - 1.64)	.72
Smoking status	Never / Current or former	1.15 (0.68 - 1.93)	.60
Duration of diabetes	＜10 years / ≥ 10 years	1.30 (0.87 - 1.96)	.21
Fasting plasma glucose	＜180 mg/dL / ≥ 180 mg/dL	0.94 (0.58 - 1.51)	.79
Systolic blood pressure	＜130 mmHg / ≥ 130 mmHg	0.88 (0.55 - 1.41)	.60
Diastolic blood pressure	＜80 mmHg / ≥ 80 mmHg	1.30 (0.83 - 2.01)	.25

© 2025 Sasako T et al. *JAMA Ophthalmology*.

LDL cholesterol	＜120 mg/dL / ≥ 120 mg/dL	1.09 (0.73 - 1.65)	.67
HDL cholesterol	＜40 mg/dL / ≥ 40 mg/dL	0.75 (0.42 - 1.33)	.33
Triglyceride	＜150 mg/dL / ≥ 150 mg/dL	1.01 (0.64 - 1.58)	.98
Urine ACR	＜30mg/g･Cr / ≥ 30mg/g･Cr	1.29 (0.86 - 1.94)	.22
eGFR	＜60mL/min/1.73m^2 / ≥ 60mL/min/1.73m^2	1.42 (0.72 - 2.78)	.31

Multivariable Cox regression for progression of retinopathy in participants with non-proliferative retinopathy at baseline with allocation factors and pre-specified factors at baseline. Covariates were available in 428 participants and 106 events were observed. The referred category is indicated in the left. CVD, cardiovascular disease; LDL, low-density lipoprotein; HDL, high-density lipoprotein; ACR, albumin-creatinine ratio; eGFR, estimated glomerular filtration rate.

© 2025 Sasako T et al. *JAMA Ophthalmology*.

eTable 6. HbA_{1c} Change During the First Year of Intervention and Hypoglycemia

HbA_{1C} change during the first year	Hypoglycemia during intervention	
	No	Yes
Reduced by 1% or more	606 (63.3%)	352 (36.7%)
Reduced by less than 1%	913 (69.0%)	411 (31.0%)

Participants were stratified by the difference between HbA_{1C} at baseline and HbA_{1C} at 1 year after randomization and the presence or absence of at least one hypoglycemic episode during the intervention period.

© 2025 Sasako T et al. *JAMA Ophthalmology*.

eTable 7. Yearly Incidence of Hypoglycemia During the Intervention Period

Parameter	Conventional (N = 1271)	Intensive (N = 1269)
Mean ± SD	0.13±0.55	0.41±1.20
Median (IQR)	0.00 (0.00)	0.00 (0.12)
None	988 (77.7%)	748 (58.9%)
0<, ≤0.5	200 (15.7%)	305 (24.0%)
0.5<, ≤1.0	45 (3.5%)	87 (6.9%)
1.0<	38 (3.0%)	129 (10.2%)

Data are N (%), unless otherwise indicated.

© 2025 Sasako T et al. *JAMA Ophthalmology*.

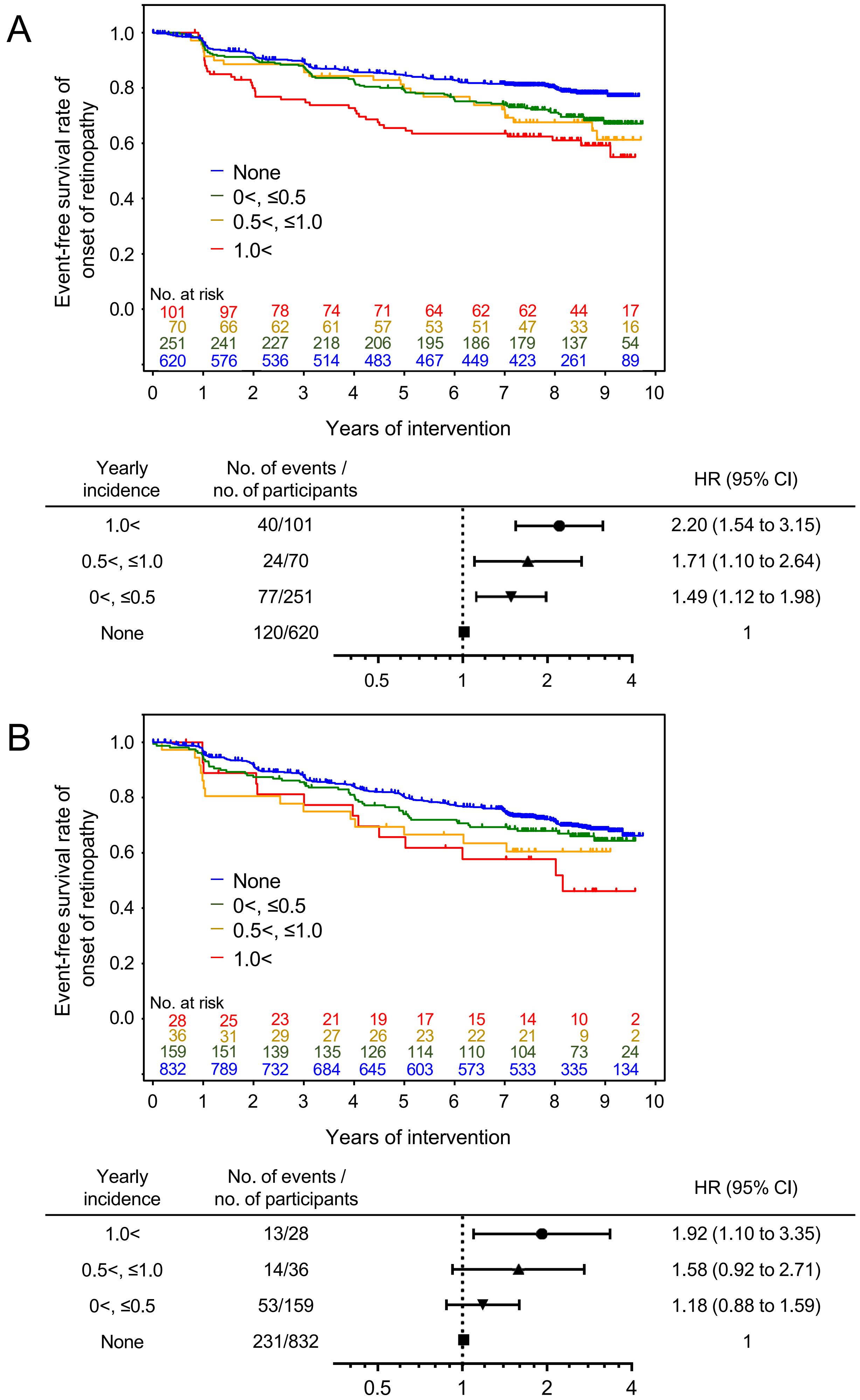

© 2025 Sasako T et al. JAMA Ophthalmology.

eFigure 1. Effects of Hypoglycemia on Onset of Retinopathy in Both Therapy Groups

(A, B) Kaplan-Meier curves and forest plots to show event-free survival rates of onset of retinopathy in, (A) the intensive therapy group, and (B) the conventional therapy group, respectively. Participants with the absence of retinopathy at baseline were included, and further stratified by the yearly incidence of hypoglycemic episodes during intervention. In total, 261 onset events were observed in 1042 participants in the intensive therapy group, and 311 onset events were observed in 1055 participants in the conventional therapy group. Bars: 95% CI.

© 2025 Sasako T et al. JAMA Ophthalmology.

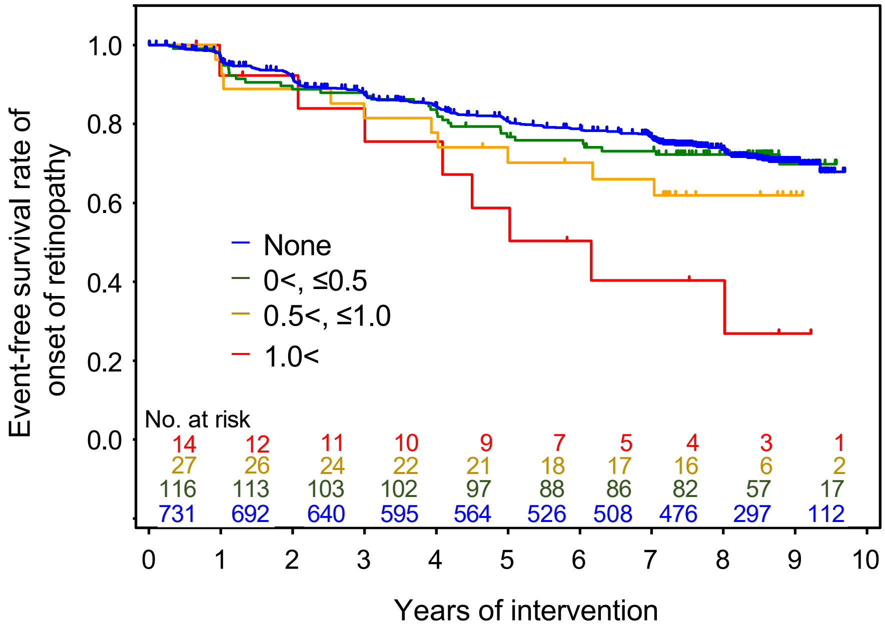

Yearly incidence	No. of events / no. of participants	HR (95% CI)
1.0<	8/14	2.94 (1.45 to 5.96)
0.5<, ≤1.0	10/27	1.52 (0.80 to 2.86)
0<, ≤0.5	33/116	1.04 (0.72 to 1.50)
None	189/731	1

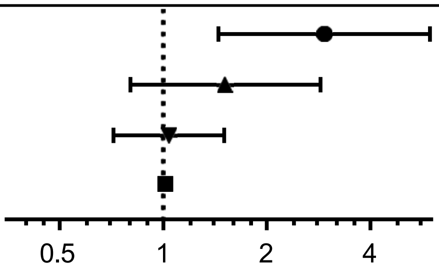

© 2025 Sasako T et al. JAMA Ophthalmology.

eFigure 2. Effects of Hypoglycemia on Onset of Retinopathy in Participants Without Receiving a Blood Glucose Meter

Kaplan-Meier curves and forest plots to show event -free survival rates of onset of retinopathy in participants without receiving a blood glucose meter, as a sensitivity analysis to examine the effect of self -monitoring of blood glucose. Participants with the absence of retinopathy at baseline were included, and we further focused on those in the conventional therapy group without history of treatment with insulin or GLP-1 receptor agonist injection, as those without receiving a blood glucose meter. In total, 240 onset events were observed in 888 participants. Bars: 95% CI.

© 2025 Sasako T et al. JAMA Ophthalmology.
