# Supplementary material for: Effect of a Multifactorial Intervention on Retinopathy in People With Type 2 Diabetes: A Secondary Analysis of the J-DOIT3 Randomized Clinical Trial
Source: JAMA Ophthalmol. 2025 Oct 23;143(12):989–97. doi: 10.1001/jamaophthalmol.2025.3819 (PMC12550739; doi:10.1001/jamaophthalmol.2025.3819)
Supplement: Supplement 3. — J-DOIT3 Study Group [file jamaophthalmol-e253819-s003.pdf]

Supplemental Online Content: Nonauthor Collaborators
*First name, last name, and suffix (if applicable) are required and will appear in PubMed.

*Group Name(s): J-DOIT3 Study Group							
***First Name and Middle Initial(s)**	***Last Name**	***Suffix (eg, Jr, III)**	Academic Degrees	Institution	Location (city, state/province, country)	Role or Contribution, eg, chair, principal investigator	Group (if more than 1 Group listed in the byline) and/or Subgroup (eg, Steering Committee)
Masakazu	Haneda		MD, PhD	Asahikawa Medical University	Asahikawa, Hokkaido	Investigator	
Yasunori	Iwashima		MD	Yoshida Hospital	Asahikawa, Hokkaido	Investigator	
Mizuho	Okada		MD	Yoshida Hospital	Asahikawa, Hokkaido	Investigator	
Toshihiro	Suda		MD, PhD	Hirosaki University Hospital	Hirosaki, Aomori	Investigator	
Naoki	Tamasawa		MD, PhD	Hirosaki University Hospital	Hirosaki, Aomori	Investigator	
Makoto	Daimon		MD, PhD	Hirosaki University Hospital	Hirosaki, Aomori	Investigator	
Jo	Satoh		MD, PhD	Iwate Medical University Hospital	Morioka, Iwate	Investigator	
Noriko	Takebe		MD, PhD	Iwate Medical University Hospital	Morioka, Iwate	Investigator	
Yasushi	Ishigaki		MD, PhD	Iwate Medical University Hospital	Morioka, Iwate	Investigator	
Tsuyoshi	Watanabe		MD, PhD	Fukushima Medical University	Fukushima, Fukushima	Investigator	
Hiroaki	Satoh		MD, PhD	Fukushima Medical University	Fukushima, Fukushima	Investigator	
Kikuo	Kasai		MD, PhD	Dokkyo Medical University	Mibu, Tochigi	Investigator	
Yoshimasa	Aso		MD, PhD	Dokkyo Medical University	Mibu, Tochigi	Investigator	
Shun	Ishibashi		MD, PhD	Jichi Medical University	Shimotsuke, Tochigi	Investigator	
Shigehiro	Katayama		MD, PhD	Saitama Medical University Hospital	Moroyama, Saitama	Investigator	
San-e	Ishikawa		MD, PhD	Jichi Medical University Saitama Medical Center	Saitama, Saitama	Investigator	
Masafumi	Kakei		MD, PhD	Jichi Medical University Saitama Medical Center	Saitama, Saitama	Investigator	
Kazuyuki	Namai		MD, PhD	Saitama Red Cross Hospital	Saitama, Saitama	Investigator	
Naotake	Hashimoto		MD, PhD	Asahi General Hospital	Asahi, Chiba	Investigator	
Yoshifumi	Suzuki		MD, PhD	Asahi General Hospital	Asahi, Chiba	Investigator	
Shunichiro	Onishi		MD, PhD	Asahi General Hospital	Asahi, Chiba	Investigator	
Koutaro	Yokote		MD, PhD	Chiba University Hospital	Chiba, Chiba	Investigator	
Masafumi	Matsuda		MD, PhD	Kameda Medical Center	Kamogawa, Chiba	Investigator	
Masahiro	Masuzawa		MD	Kameda Medical Center	Kamogawa, Chiba	Investigator	
Mitsutaka	Motoyoshi		MD, PhD	Memorial Hospital of Kazusa	Kisarazu, Chiba	Investigator	
Yoichi	Hayashi		MD, PhD	Nihon University Itabashi Hospital	Itabashi, Tokyo	Investigator	

Supplemental Online Content: Nonauthor Collaborators
*First name, last name, and suffix (if applicable) are required and will appear in PubMed.

***First Name and Middle Initial(s)**	***Last Name**	***Suffix (eg, Jr, III)**	Academic Degrees	Institution	Location (city, state/province, country)	Role or Contribution, eg, chair, principal investigator	Group (if more than 1 Group listed in the byline) and/or Subgroup (eg, Steering Committee)
Satoshi	Saito		MD, PhD	Nihon University Itabashi Hospital	Itabashi, Tokyo	Investigator	
Norikazu	Ogihara		MD, PhD	Nihon University Itabashi Hospital	Itabashi, Tokyo	Investigator	
Hisamitsu	Ishihara		MD, PhD	Nihon University Itabashi Hospital	Itabashi, Tokyo	Investigator	
Naoko	Tajima		MD, PhD, Master of Hygiene	The Jikei University School of Medicine	Minato, Tokyo	Investigator	
Kazunori	Utsunomiya		MD, PhD	The Jikei University School of Medicine	Minato, Tokyo	Investigator	
Akira	Shimada		MD, PhD	Keio University School of Medicine	Shinjuku, Tokyo	Investigator	
Hiroshi	Itoh		MD, PhD	Keio University School of Medicine	Shinjuku, Tokyo	Investigator	
Toru	Hiyoshi		MD, PhD	Japanese Red Cross Medical Center	Shibuya, Tokyo	Investigator	
Ryuzo	Kawamori		MD, PhD	Juntendo University Graduate School of Medicine	Bunkyo, Tokyo	Investigator	
Hirotaka	Watada		MD, PhD	Juntendo University Graduate School of Medicine	Bunkyo, Tokyo	Investigator	
Michio	Hayashi		MD, PhD	NTT Medical Center Tokyo	Shinagawa, Tokyo	Investigator	
Yasumichi	Mori		MD, PhD	Federation of National Public Service Personnel Mutual Aid Associations, Toranomon Hospital	Minato, Tokyo	Investigator	
Teruo	Shiba		MD, PhD	Mitsui Memorial Hospital	Chiyoda, Tokyo	Investigator	
Akihiro	Isogawa		MD, PhD	Mitsui Memorial Hospital	Chiyoda, Tokyo	Investigator	
Hiroshi	Sakura		MD, PhD	Tokyo Women's Medical University Hospital	Shinjuku, Tokyo	Investigator	
Yasuko	Uchigata		MD, PhD	Tokyo Women's Medical University Hospital	Shinjuku, Tokyo	Investigator	
Masato	Odawara		MD, PhD	Tokyo Medical University Hospital	Shinjuku, Tokyo	Investigator	
Kazuyuki	Tobe		MD, PhD	The University of Tokyo Hospital	Bunkyo, Tokyo	Investigator	
Kazuhisa	Tsukamoto		MD, PhD	The University of Tokyo Hospital	Bunkyo, Tokyo	Investigator	

Supplemental Online Content: Nonauthor Collaborators
*First name, last name, and suffix (if applicable) are required and will appear in PubMed.

***First Name and Middle Initial(s)**	***Last Name**	***Suffix (eg, Jr, III)**	Academic Degrees	Institution	Location (city, state/province, country)	Role or Contribution, eg, chair, principal investigator	Group (if more than 1 Group listed in the byline) and/or Subgroup (eg, Steering Committee)
Toshimasa	Yamauchi		MD, PhD	The University of Tokyo Hospital	Bunkyo, Tokyo	Investigator	
Tamio	Teramoto		MD, PhD	Teikyo University School of Medicine Hospital	Itabashi, Tokyo	Investigator	
Yukio	Hirata		MD, PhD	Tokyo Medical and Dental University, University Hospital of Medicine	Bunkyo, Tokyo	Investigator	
Isao	Uchimura		MD, PhD	Tokyo Medical and Dental University, University Hospital of Medicine	Bunkyo, Tokyo	Investigator	
Yoshihiro	Ogawa		MD, PhD	Tokyo Medical and Dental University, University Hospital of Medicine	Bunkyo, Tokyo	Investigator	
Gen	Yoshino		MD, PhD	Toho University Medical Center, Ohmori Hospital	Ota, Tokyo	Investigator	
Takahisa	Hirose		MD, PhD	Toho University Medical Center, Ohmori Hospital	Ota, Tokyo	Investigator	
Hiroshi	Kajio		MD, PhD	National Center for Global Health and Medicine Hospital	Shinjuku, Tokyo	Investigator	
Yoshihito	Atsumi		MD	Saiseikai Central Hospital	Minato, Tokyo	Investigator	
Akira	Shimada		MD, PhD	Saiseikai Central Hospital	Minato, Tokyo	Investigator	
Yoichi	Oikawa		MD, PhD	Saiseikai Central Hospital	Minato, Tokyo	Investigator	
Atsushi	Araki		MD, PhD	Tokyo Metropolitan Geriatric Hospital	Itabashi, Tokyo	Investigator	
Akio	Ueki		MD, PhD	Hachioji Medical Center of Tokyo Medical University	Hachioji, Tokyo	Investigator	
Atsushi	Ohno		MD, PhD	Hachioji Medical Center of Tokyo Medical University	Hachioji, Tokyo	Investigator	
Masafumi	Kitaoka		MD, PhD	Showa General Hospital	Kodaira, Tokyo	Investigator	
Yoshikuni	Fujita		MD, PhD	Kitasato University Hospital	Sagamihara, Kanagawa	Investigator	
Tatsumi	Moriya		MD, PhD	Kitasato University Hospital	Sagamihara, Kanagawa	Investigator	
Taiki	Tojo		MD, PhD	Kitasato University Hospital	Sagamihara, Kanagawa	Investigator	

Supplemental Online Content: Nonauthor Collaborators
*First name, last name, and suffix (if applicable) are required and will appear in PubMed.

***First Name and Middle Initial(s)**	***Last Name**	***Suffix (eg, Jr, III)**	Academic Degrees	Institution	Location (city, state/province, country)	Role or Contribution, eg, chair, principal investigator	Group (if more than 1 Group listed in the byline) and/or Subgroup (eg, Steering Committee)
Masayoshi	Shichiri		MD, PhD	Kitasato University Hospital	Sagamihara, Kanagawa	Investigator	
Yasushi	Tanaka		MD, PhD	St.Marianna University School of Medicine	Kawasaki, Kanagawa	Investigator	
Daisuke	Suzuki		MD, PhD	Tokai University School of Medicine	Isehara, Kanagawa	Investigator	
Masao	Toyoda		MD, PhD	Tokai University School of Medicine	Isehara, Kanagawa	Investigator	
Kumiko	Hamano		MD	Shonan Kamakura General Hospital	Kamakura, Kanagawa	Investigator	
Rieko	Komi		MD	Shonan Kamakura General Hospital	Kamakura, Kanagawa	Investigator	
Yasuo	Terauchi		MD, PhD	Yokohama City University Hospital	Yokohama, Kanagawa	Investigator	
Nobuaki	Kuzuya		MD	Yokohama Sakae Kyosai Hospital, Federation of National Public Service Personnel Mutual Associations	Yokohama, Kanagawa	Investigator	
Masayo	Yamada		MD, PhD	Yokohama Sakae Kyosai Hospital, Federation of National Public Service Personnel Mutual Associations	Yokohama, Kanagawa	Investigator	
Tadahisa	Momoki		MD	Odawara Municipal Hospital	Odawara, Kanagawa	Investigator	
Koichiro	Sato		MD, PhD	Odawara Municipal Hospital	Odawara, Kanagawa	Investigator	
Mitsuo	Imura		MD, PhD	Yaizu City Hospital	Yaizu, Shizuoka	Investigator	
Toshinari	Takamura		MD, PhD	Kanazawa University Hospital	Kanazawa, Ishikawa	Investigator	
Hiroaki	Yamamoto		MD	Nagano Chuo Hospital	Nagano, Nagano	Investigator	
Hiroshi	Sobajima		MD, PhD	Ogaki Municipal Hospital	Ogaki, Gifu	Investigator	
Akihiko	Yoneyama		MD, PhD	Gamagori City Hospital	Gamagori, Aichi	Investigator	
Kenichi	Ito		MD, PhD	Gamagori City Hospital	Gamagori, Aichi	Investigator	
Hiroshi	Tanaka		MD, PhD	Chukyo Hospital	Nagoya, Aichi	Investigator	
Masayuki	Hayashi		MD, PhD	Chukyo Hospital	Nagoya, Aichi	Investigator	
Fumiko	Yamakawa		MD	Chukyo Hospital	Nagoya, Aichi	Investigator	

Supplemental Online Content: Nonauthor Collaborators
*First name, last name, and suffix (if applicable) are required and will appear in PubMed.

***First Name and Middle Initial(s)**	***Last Name**	***Suffix (eg, Jr, III)**	Academic Degrees	Institution	Location (city, state/province, country)	Role or Contribution, eg, chair, principal investigator	Group (if more than 1 Group listed in the byline) and/or Subgroup (eg, Steering Committee)
Yasuhisa	Kato		MD, PhD	Nagoya Medical Center	Nagoya, Aichi	Investigator	
Mitsuyasu	Itoh		MD, PhD	Fujita Health University Hospital	Toyoake, Aichi	Investigator	
Atsushi	Suzuki		MD, PhD	Fujita Health University Hospital	Toyoake, Aichi	Investigator	
Mikihiro	Nakayama		MD, PhD	Chubu Rosai Hospital	Nagoya, Aichi	Investigator	
Takahisa	Sano		MD	Chubu Rosai Hospital	Nagoya, Aichi	Investigator	
Eitaro	Nakashima		MD, PhD	Chubu Rosai Hospital	Nagoya, Aichi	Investigator	
Yasuhiro	Sumida		MD, PhD	Mie University Hospital	Tsu, Mie	Investigator	
Yutaka	Yano		MD, PhD	Mie University Hospital	Tsu, Mie	Investigator	
Tsuyoshi	Tanaka		MD, PhD	National Hospital Organization Mie Chuo Medical Center	Tsu, Mie	Investigator	
Kazuya	Murata		MD, PhD	Ise Red Cross Hospital	Ise, Mie	Investigator	
Atsunori	Kashiwagi		MD, PhD	Shiga University of Medical Science	Otsu, Shiga	Investigator	
Hiroshi	Maegawa		MD, PhD	Shiga University of Medical Science	Otsu, Shiga	Investigator	
Shigeo	Kono		MD, PhD	National Hospital Organization Kyoto Medical Center	Kyoto, Kyoto	Investigator	
Nobuya	Inagaki		MD, PhD	Kyoto University Hospital	Kyoto, Kyoto	Investigator	
Keisuke	Kosugi		MD, PhD	Osaka Police Hospital	Osaka, Osaka	Investigator	
Tetsuyuki	Yasuda		MD, PhD	Osaka Police Hospital	Osaka, Osaka	Investigator	
Yasunao	Yoshimasa		MD, PhD	National Cerebral and Cardiovascular Center	Suita, Osaka	Investigator	
Ichiro	Kishimoto		MD, PhD	National Cerebral and Cardiovascular Center	Suita, Osaka	Investigator	
Toshihiko	Sato		MD, PhD	Osaka City General Hospital	Osaka, Osaka	Investigator	
Masayuki	Hosoi		MD, PhD	Osaka City General Hospital	Osaka, Osaka	Investigator	
Tomoyuki	Yamasaki		MD, PhD	Osaka Medical Center for Cancer and Cardiovascular Diseases	Osaka, Osaka	Investigator	
Munehide	Matsuhisa		MD, PhD	Osaka University Hospital	Suita, Osaka	Investigator	
Iichiro	Shimomura		MD, PhD	Osaka University Hospital	Suita, Osaka	Investigator	
Ataru	Taniguchi		MD, PhD	Kansai Electric Power Hospital	Osaka, Osaka	Investigator	

Supplemental Online Content: Nonauthor Collaborators
*First name, last name, and suffix (if applicable) are required and will appear in PubMed.

***First Name and Middle Initial(s)**	***Last Name**	***Suffix (eg, Jr, III)**	Academic Degrees	Institution	Location (city, state/province, country)	Role or Contribution, eg, chair, principal investigator	Group (if more than 1 Group listed in the byline) and/or Subgroup (eg, Steering Committee)
Akira	Kuroe		MD, PhD	Kansai Electric Power Hospital	Osaka, Osaka	Investigator	
Takeshi	Kurose		MD, PhD	Kansai Electric Power Hospital	Osaka, Osaka	Investigator	
Hiroshi	Ikegami		MD, PhD	Kindai University Faculty of Medicine	Osakasayama, Osaka	Investigator	
Takeshi	Ohara		MD, PhD	Kobe University Graduate School of Medicine	Kobe, Hyogo	Investigator	
Kazuhiko	Sakaguchi		MD, PhD	Kobe University Graduate School of Medicine	Kobe, Hyogo	Investigator	
Mitsuyoshi	Namba		MD, PhD	Hyogo College of Medicine College Hospital	Nishinomiya, Hyogo	Investigator	
Masaki	Ikeda		MD, PhD	Seimeikai Ikeda Hospital	Amagasaki, Hyogo	Investigator	
Hiroki	Ikeda		MD, PhD	Seimeikai Ikeda Hospital	Amagasaki, Hyogo	Investigator	
Kohei	Kaku		MD, PhD	Kawasaki Medical School Hospital	Kurashiki, Okayama	Investigator	
Kenji	Takahashi		MD, PhD	Kurashiki Central Hospital	Kurashiki, Okayama	Investigator	
Hirofumi	Makino		MD, PhD	Okayama University Hospital	Okayama, Okayama	Investigator	
Masazumi	Fujiwara		MD	Saijo Central Hospital	Saijo, Ehime	Investigator	
Ikki	Shimizu		MD, PhD	Ehime Prefectural Central Hospital	Matsuyama, Ehime	Investigator	
Keizo	Ono		MD	Ehime Prefectural Central Hospital	Matsuyama, Ehime	Investigator	
Osamu	Ebisui		MD, PhD	Ehime Prefectural Central Hospital	Matsuyama, Ehime	Investigator	
Yukio	Tanizawa		MD, PhD	Yamaguchi University Hospital	Ube, Yamaguchi	Investigator	
Yosuke	Okada		MD, PhD	University of Occupational and Environmental Health	Kitakyushu, Fukuoka	Investigator	
Shoichi	Natori		MD	Iizuka Hospital	Iizuka, Fukuoka	Investigator	
Takehiko	Kodera		MD	Iizuka Hospital	Iizuka, Fukuoka	Investigator	
Naoichi	Sato		MD	Iizuka Hospital	Iizuka, Fukuoka	Investigator	
Makoto	Ide		MD	Iizuka Hospital	Iizuka, Fukuoka	Investigator	
Kentaro	Yamada		MD, PhD	Kurume University Hospital	Kurume, Fukuoka	Investigator	
Yuji	Tajiri		MD, PhD	Kurume University Hospital	Kurume, Fukuoka	Investigator	

Supplemental Online Content: Nonauthor Collaborators
*First name, last name, and suffix (if applicable) are required and will appear in PubMed.

***First Name and Middle Initial(s)**	***Last Name**	***Suffix (eg, Jr, III)**	Academic Degrees	Institution	Location (city, state/province, country)	Role or Contribution, eg, chair, principal investigator	Group (if more than 1 Group listed in the byline) and/or Subgroup (eg, Steering Committee)
Fumio	Umeda		MD	Fukuoka City Medical Association Hospital	Fukuoka, Fukuoka	Investigator	
Shoichi	Natori		MD	Fukuoka City Medical Association Hospital	Fukuoka, Fukuoka	Investigator	
Tomoaki	Eto		MD	Fukuoka City Medical Association Hospital	Fukuoka, Fukuoka	Investigator	
Kazuo	Mimura		MD	Fukuoka City Medical Association Hospital	Fukuoka, Fukuoka	Investigator	
Shinsuke	Hiramatsu		MD	Fukuoka City Medical Association Hospital	Fukuoka, Fukuoka	Investigator	
Tomoaki	Inoue		MD, PhD	Fukuoka City Medical Association Hospital	Fukuoka, Fukuoka	Investigator	
Ryoko	Takei		MD	Fukuoka City Medical Association Hospital	Fukuoka, Fukuoka	Investigator	
Atsushi	Ogo		MD, PhD	National Hospital Organization Kyushu Medical Center	Fukuoka, Fukuoka	Investigator	
Katsumi	Eguchi		MD, PhD	Nagasaki University Hospital	Nagasaki, Nagasaki	Investigator	
Eiji	Kawasaki		MD, PhD	Nagasaki University Hospital	Nagasaki, Nagasaki	Investigator	
Yuji	Koide		MD, PhD	Nagasaki University Hospital	Nagasaki, Nagasaki	Investigator	
Eiichi	Araki		MD, PhD	Kumamoto University Hospital	Kumamoto, Kumamoto	Investigator	
Hideaki	Jinnouchi		MD, PhD	Jinnouchi Hospital Diabetes Care Center	Kumamoto, Kumamoto	Investigator	
Yasuo	Ueda		MD, PhD	The Japanese Red Cross Nagasaki Genbaku Hospital	Nagasaki, Nagasaki	Investigator	
Masamitsu	Nakazato		MD, PhD	University of Miyazaki Hospital	Miyazaki, Miyazaki	Investigator	
