# Supplementary material for: Effect of a Multifactorial Intervention on Retinopathy in People With Type 2 Diabetes: A Secondary Analysis of the J-DOIT3 Randomized Clinical Trial
Source: JAMA Ophthalmol. 2025 Oct 23;143(12):989–97. doi: 10.1001/jamaophthalmol.2025.3819 (PMC12550739; doi:10.1001/jamaophthalmol.2025.3819)
Supplement: Supplement 4. — Data Sharing Statement [file jamaophthalmol-e253819-s004.pdf]

Data Sharing Statement

Sasako. Effect of a Multifactorial Intervention on Retinopathy in People With Type 2 Diabetes. *JAMA Ophthalmol*. Published October 23, 2025. doi:10.1001/jamaophthalmol.2025.3819

Data
Additional Information: ClinicalTrials.gov Identifier; https://clinicaltrials.gov/study/NCT00300976; NCT00300976.
Data available: No
